# *slc26a12*—A novel member of the *slc26* family, is located in tandem with *slc26a2* in coelacanths, amphibians, reptiles, and birds

**DOI:** 10.14814/phy2.16089

**Published:** 2024-06-03

**Authors:** Ayumi Nagashima, Kota Torii, Chihiro Ota, Akira Kato

**Affiliations:** ^1^ School of Life Science and Technology Tokyo Institute of Technology Yokohama Japan

**Keywords:** evolution, gene loss, pseudogenization, Slc26a12, Slc26a2

## Abstract

Solute carrier family 26 (Slc26) is a family of anion exchangers with 11 members in mammals (named Slc26a1‐a11). Here, we identified a novel member of the *slc26* family, *slc26a12*, located in tandem with *slc26a2* in the genomes of several vertebrate lineages. BLAST and synteny analyses of various jawed vertebrate genome databases revealed that *slc26a12* is present in coelacanths, amphibians, reptiles, and birds but not in cartilaginous fishes, lungfish, mammals, or ray‐finned fishes. In some avian and reptilian lineages such as owls, penguins, egrets, and ducks, and most turtles examined, *slc26a12* was lost or pseudogenized. Phylogenetic analysis showed that Slc26a12 formed an independent branch with the other Slc26 members and Slc26a12, Slc26a1 and Slc26a2 formed a single branch, suggesting that these three members formed a subfamily in Slc26. In jawless fish, hagfish have two genes homologous to *slc26a2* and *slc26a12*, whereas lamprey has a single gene homologous to *slc26a2*. African clawed frogs express *slc26a12* in larval gills, skin, and fins. These results show that *slc26a12* was present at least before the separation of lobe‐finned fish and tetrapods; the name *slc26a12* is appropriate because the gene duplication occurred in the distant past.

## 1 INTRODUCTION

In humans, membrane transport proteins comprise a large group, accounting for ~10% of all protein‐coding genes, and their nomenclature is systematically organized and numbered (Hediger et al., 2013). Recently, with the development of next‐generation sequencing, the genomes of many vertebrate species have been sequenced and published (Koepfli et al., 2015). Comparative genomic analyses using these databases have shown that the genetic composition of jawed vertebrates is relatively conserved and that each species has lineage‐ and species‐specific gene gain and loss. Consequently, some genes that are widely present in vertebrates but absent in some lineages, including humans, have been reported and named as family members with new numbers. These genes consist of the following two types: (i) old genes that were present in the ancestral species but were lost in lineages including mammals and (ii) new genes that were specifically gained in nonmammalian lineages. For example, the human genome contains 13 genes that encode aquaporin (Aqp) water channels *aqp0*–*aqp12*. Vertebrate Aqps are distributed in 17 subfamilies (Aqp0–16) (Finn et al., 2014); therefore, humans do not have four *aqp* genes, *aqp13*–*aqp16* (Chauvigne et al., 2019; Finn et al., 2014) (Note that, in this article, protein name abbreviations of all species are shown with the first letter capitalized, and gene names of all species are shown as lowercase and italicized). Molecular phylogenetic analysis of the solute carrier family 1 (Slc1) of amino acid transporters identified new members, Slc1a8 and Slc1a9, that are not present in mammalian lineages (Gesemann et al., 2010). Large‐scale analysis of the glucose transporter family Slc2 revealed Slc2a15–a20, where Slc2a15 is widely distributed in birds, reptiles, amphibians, and teleosts, and Slc2a18‐a20 is present in teleosts (Xiong and Lei, 2021). Slc8a4, which encodes Na^+^‐Ca^2+^ exchanger 4 (Ncx4), is found in teleosts, amphibians, and reptiles (Spencer et al., 2020). Nine members of the Slc12 family, Slc12a1–a9, are known in mammals (Arroyo et al., 2013, Xiong and Lei, 2021). *slc12a10*, which encodes the Na^+^‐Cl^−^ cotransporter 2 (Ncc2), was originally found in fish (Cutler and Cramb, 2008, Hiroi et al., 2008, Wang et al., 2009) and has also been found in amphibians, non‐avian reptiles, and some mammals such as horses (Motoshima et al., 2023). A pseudogene for *slc12a10* at the same chromosomal locus has been identified in various mammalian genome databases. The human urea transporter family consists of Slc14a1 (Ut‐b) and Slc14a2 (Ut‐a), and Ut‐c have been found in teleosts, and Ut‐3, similar to Ut‐c, in cartilaginous fish (Kakumura et al., 2009, Mistry et al., 2005).

Slc26 is a family of anion exchangers, and 11 members, Slc26a1–11, are present in mammals. Slc26 proteins share 12 transmembrane regions and a sulfate transporter anti‐sigma factor antagonist (STAS) domain in the intracellular carboxy‐terminal region (Alper and Sharma, 2013, Mount and Romero, 2004). These transport anions such as Cl^−^, bicarbonate, sulfate, formate, and oxalate ions. The Slc26a1 protein was first identified as a Na^+^‐independent sulfate transporter and is expressed on the plasma membrane of the liver and renal tubules (Bissig et al., 1994). Whole‐exome sequencing of a patient presenting with painful perichondritis, hyposulfatemia, and renal sulfate wasting revealed a homozygous mutation in the human Slc26a1 gene (*slc26a1*), and Slc26a1 activity is a major determinant of sulfate homeostasis in humans (Pfau et al., 2023). Slc26a2 was initially isolated by positional cloning of diastrophic dysplasia and is also called diastrophic dysplasia sulfate transporter (Dtdst) (Hastbacka et al., 1994). Numerous *slc26a2* mutations have been identified in human recessive chondrodysplasia syndromes (Alper and Sharma, 2013, Jackson et al., 2012). Analysis of mice expressing Slc26a2 mutants has revealed skeletal abnormalities, decreased chondrocyte proliferative activity, and decreased sulfate absorption into chondrocytes (Alper and Sharma, 2013, Forlino et al., 2005). Slc26a3 was found to be downregulated in adenoma (Dra), and is localized on the apical membrane of intestinal epithelial cells (Schweinfest et al., 1993). It is also responsible for electroneutral Cl^−^ absorption and HCO_3_^−^ secretion in the intestine (Schweinfest et al., 2006). Slc26a4, also called pendrin, is mutated in approximately half of the patients with Pendred syndrome (Coyle et al., 1996, Sheffield et al., 1996). Slc26a5, also known as prestin, is expressed in the outer hair cells of the cochlea and contributes to the auditory function (Zheng et al., 2000). Slc26a6, also known by the name putative anion transporter‐1 (Pat‐1) or chloride/formate exchanger (Cfex), contributes to anion metabolism in the intestine and kidney, intestinal oxalate excretion, and regulation of cystic fibrosis transmembrane conductance regulator (Cftr) and pancreatic duct HCO_3_^−^ secretion (Wang et al., 2005, Lohi et al., 2000, Knauf et al., 2001).

Slc26 has also been reported in fish, insects, and corals and is widely present in the animal kingdom, contributing to its anion metabolism. In fish, Slc26 members contribute to renal sulfate efflux in seawater fish (Kato et al., 2009, Kato and Watanabe, 2016, Katoh et al., 2006, Watanabe and Takei, 2011), Cl^−^ uptake via the gill in freshwater fish (Bayaa et al., 2009, Hwang et al., 2011, Perry et al., 2009), intestinal bicarbonate secretion in seawater fish (Ando et al., 2014, Genz et al., 2011, Kurita et al., 2008, Ruhr et al., 2015), renal sulfate reabsorption in freshwater fish (Nakada et al., 2005), and intestinal oxalate excretion (Whittamore, 2020). In insects such as *Anopheles*, Slc26 contributes to anion metabolism in the gut and Malpighian tubes (Hirata, Czapar et al., 2012; Hirata, Cabrero et al., 2012). Corals have three types of Slc26: α, β, and γ (Zoccola et al., 2015). In a comparative analysis of the Slc26 family in vertebrates, we initially found an uncharacterized *slc26* homolog present in tandem with *slc26a2* in the genome databases of chickens, *Xenopus*, and coelacanths. In this study, we performed molecular phylogenetic and synteny analyses, and showed that the divergence between the new *slc26* gene and other known *slc26* genes is ancient. We propose the name *slc26a12* for this novel member of the Slc26 family.

## 2 MATERIALS AND METHODS

### 2.1 Identification of *slc26a12* in vertebrate genome databases

*slc26a12* was initially identified in coelacanth, western clawed frog, green anole, and chicken as an uncharacterized *slc26* gene located in tandem with *slc26a2* using genome databases (Table 1). The predicted amino acid sequences and accession numbers of Slc26a1/a2/a12 were obtained from these databases and used as queries for BLAST analysis to determine the presence or absence of Slc26a1/a2/a12 in each vertebrate species. BLASTP and TBLASTN analyses were performed on protein or whole‐genome shotgun databases of the 59 vertebrate species listed in Table 1 using the NCBI BLAST server (https://blast.ncbi.nlm.nih.gov) or Ensembl genome browser (https://www.ensembl.org) (Martin et al., 2023), and the amino acid sequences and accession numbers of Slc26a1/a2/a12 in each species were collected.

**TABLE 1:** Accession numbers of *slc26a1*, *slc26a2*, and *slc26a12* in various vertebrate species.

Species	Genome database	Reference	*slc26a1*	*slc26a2*	*slc26a12*
Human (*Homo sapiens*)	GCF_000001405.40	(Lander et al., 2001)	NM_022042.4	NM_000112.4	Not present
Dog (*Canis lupus familiaris*)	GCF_014441545.1	(Wang et al., 2021)	XM_038662138.1	XM_038663660.1	Not present
Horse (*Equus caballus*)	GCF_002863925.1	(Kalbfleisch et al., 2018)	XM_014738677.2	NM_001081934.2	Not present
Nine‐banded armadillo (*Dasypus novemcinctus*)	GCF_030445035.1	Vertebrate genomes project	XM_058301884.1	XM_058280953.1	Not present
African savanna elephant (*Loxodonta africana*)	GCF_000001905.1	Broad Institute	XM_010591446.2	XM_023552693.1	Not present
Tasmanian devil (*Sarcophilus harrisii*)	GCF_902635505.1	Wellcome Sanger Institute	XM_023506152.2	XM_031953507.1	Not present
Gray short‐tailed opossum (*Monodelphis domestica*)	GCF_027887165.1	(Mikkelsen et al., 2007)	XM_007496877.3	XM_007473862.3	Not present
Platypus (*Ornithorhynchus anatinus*)	GCF_004115215.2	(Zhou et al., 2021)	XM_007672822.4	XM_007673322.3	Not present
Bengalese finch (*Lonchura striata*)	GCF_005870125.1	(Colquitt et al., 2018)	XM_021547989.2	XM_021547182.2	XM_021547186.2
Burrowing owl (*Athene cunicularia*)	GCF_003259725.1	(Mueller et al., 2018)	XM_026866180.1	XM_026858357.1	Pseudogene
Dalmatian pelican (*Pelecanus crispus*)	GCF_000687375.1	(Zhang et al., 2014)	XM_009487553.1	XM_009488051.1	XM_009488053.1
Little egret (*Egretta garzetta*)	GCF_000687185.1	(Zhang et al., 2014)	XM_009639005.2	XM_009647084.2	Not present
Emperor penguin (*Aptenodytes forsteri*)	GCF_000699145.1	(Zhang et al., 2014)	XM_009274646.2	XM_009285731.2	Pseudogene
Adelie penguin (*Pygoscelis adeliae*)	GCF_000699145.1	(Zhang et al., 2014)	XM_009274646.2	XM_009285731.2	Pseudogene
Northern fulmar (*Fulmarus glacialis*)	GCF_000690835.1	(Zhang et al., 2014)	KFV96290 (part)	XM_009586189.1	XM_009581236.1
Golden eagle (*Aquila chrysaetos*)	GCF_900496995.4	Wellcome Sanger Institute	XM_030003808.2	XM_029997775.1	ENSACCT00020017627.1
Ruff (*Calidris pugnax*)	GCF_001431845.1		XM_014958158.1	XM_014948211.1	ENSCPUT00000013074.1
Mallard (*Anas platyrhynchos*)	GCF_015476345.1	(Li et al., 2021)	XM_038170758.1	XM_027465502.2	Pseudogene
Chicken (*Gallus gallus*)	GCF_016699485.2	(Smith et al., 2022)	XM_004949339.5	NM_001389738.2	XM_046900518.1
African ostrich (*Struthio camelus*)	GCF_000698965.1	(Zhang et al., 2014)	XM_009687577.1	XM_009685623.1	XM_009685695.1
American alligator (*Alligator mississippiensis*)	GCF_030867095.1		XM_006259257.3	XM_014597637.3	XM_059733419.1
Red‐eared slider turtle (*Trachemys scripta*)	GCF_013100865.1	(Brian Simison et al., 2020)	XM_034774757.1	XM_034780206.1	Not present
Mexican gopher tortoise (*Gopherus flavomarginatus*)	GCF_025201925.1		XM_050943084.1	XM_050962641.1	Not present
Green sea turtle (*Chelonia mydas*)	GCF_015237465.2	(Bentley et al., 2023)	XM_007056848.3	XM_007057263.4	Pseudogene
Common snapping turtle (*Chelydra serpentina*)	GCA_018859375.1	(Das et al., 2020)	JAHGAV010000666.1	JAHGAV010000001.1	ENSCSRT00000017774.1
Chinese soft‐shelled turtle (*Pelodiscus sinensis*)	GCF_000230535.1	(Wang et al., 2013)	XM_006135143.3	XM_006138099.3	Not Present
Green anole (*Anolis carolinensis*)	GCF_000090745.2	(Alfoldi et al., 2011)	XM_008114587.2	XM_003217337.3	XM_003217349.3
Japanese gecko (*Gekko japonicus*)	GCF_001447785.1	(Liu et al., 2015)	XM_015417411.1	XM_015419851.1	XM_015405630.1
Komodo dragon (*Varanus komodoensis*)	GCF_004798865.1	(Lind et al., 2019)	XM_044444012.1	XM_044454627.1	ENSVKKT00000019631.1
Mainland tiger snake (*Notechis scutatus*)	GCF_900518725.1	University of New South Wales, Edwards Lab	XM_026672985.1	XM_026688476.1	XM_026685280.1
Eastern brown snake (*Pseudonaja textilis*)	GCF_900518735.1	University of New South Wales, Edwards Lab	XM_026712716.1	XM_026696354.1	ENSPTXT00000013944.1
Common toad (*Bufo bufo*)	GCF_905171765.1	Wellcome Sanger Institute	XM_040418031.1	XM_040406551.1	XM_040406550.1
Western clawed frog (*Xenopus tropicalis*)	GCF_000004195.4	(Hellsten et al., 2010)	XM_002935465.3	XM_002943149.5	XM_012959807.2
Iberian ribbed newt (*Pleurodeles waltl*)	GCA_026652325.1	bioRxiv DOI:10.1101/2022.10.19.512763	KAJ1219281.1	KAJ1126660.1	KAJ1126657.1, KAJ1126654.1
Gaboon caecilian (*Geotrypetes seraphini*)	GCF_902459505.1	Wellcome Sanger Institute	XM_033916606.1	XM_033926891.1	XM_033926890.1
Tiny cayenne caecilian (*Microcaecilia unicolor*)fa	GCF_901765095.1	Wellcome Sanger Institute	XM_030194462.1	XM_030212656.1	XM_030212135.1
Two‐lined caecilian (*Rhinatrema bivittatum*)	GCA_901001135.2	Wellcome Sanger Institute	XM_029602309.1	XM_029583273.1	XM_029583269.1
West african lungfish (*Protopterus annectens*)	GCF_019279795.1	(Wang et al., 2021)	XM_044061139.1	XM_044069214.1	Not Present
Coelacanth (*Latimeria chalumnae*)	GCF_000225785.1	(Amemiya et al., 2013)	XM_006011442.1	XM_014493156.1	XM_006003789.1
Japanese pufferfish (*Takifugu rubripes*)	GCF_901000725.2	(Aparicio et al., 2002)	XM_029838056.1	XM_003978227.3	Not Present
Three‐spined stickleback (*Gasterosteus aculeatus*)	GCF_016920845.1	(Nath et al., 2021, Jones et al., 2012)	XM_040197011.1	XM_040175264.1	Not present
Nile tilapia (*Oreochromis niloticus*)	GCF_001858045.2	(Conte et al., 2017)	XM_005451404.4	XM_019355496.2	Not present
Japanese medaka (*Oryzias latipes*)	GCF_002234675.1	(Kasahara et al., 2007)	XM_011481933.3	XM_011480560.2	Not present
Greater amberjack (*Seriola dumerili*)	GCF_002260705.1	(Araki et al., 2018)	XM_022766574.1	XM_022760335.1	Not present
Atlantic cod (*Gadus morhua*)	GCF_902167405.1	(Howe et al., 2013)	XM_030341376.1	XM_030368538.1	Not present
Northern pike (*Esox lucius*)	GCF_011004845.1	(Rondeau et al., 2014)	XM_010877286.4	XM_010898954.5	Not present
Channel catfish (*Ictalurus punctatus*)	GCF_001660625.3	(Waldbieser et al., 2023, Liu et al., 2016)	XM_017493276.3	XM_017475076.3	Not present
Zebrafish (*Danio rerio*)	GCF_000002035.6	(Howe et al., 2013)	XM_005161206.4	XM_680022.5	Not present
Asian arowana (*Scleropages formosus*)	GCF_900964775.1	(Bian et al., 2016)	XM_029252658.1	XM_018733194.2	Not present
European eel (*Anguilla anguilla*)	GCF_013347855.1		XM_035389685.1	XM_035410385.1	Not present
Tarpon (*Megalops atlanticus*)	GCF_013368585.1	Vertebrate genomes project	KAG7476665.1	KAG7465003.1	Not present
Spotted gar (*Lepisosteus oculatus*)	GCF_000242695.1	(Braasch et al., 2016)	XM_006627158.2	XM_006632082.2	Not present
Sterlet (*Acipenser ruthenus*)	GCF_902713425.1	Wellcome Sanger Institute	XM_034015637.3	XM_058997183.1	Not present
Gray bichir (*Polypterus senegalus*)	GCF_016835505.1	(Bi et al., 2021)	XM_039750381.1	XM_039774862.1	Not present
Smaller spotted catshark (*Scyliorhinus canicula*)	GCF_902713615.1	Wellcome Sanger Institute	XM_038792328.1	XM_038795555.1	Not present
Little skate (*Leucoraja erinacea*)	GCF_028641065.1	(Marletaz et al., 2023)	XM_055646986.1	XM_055643019.1	Not present
Elephant shark (*Callorhinchus milii*)	GCF_018977255.1	(Venkatesh et al., 2014)	XM_007907698.2	XM_007912295.2	Not present
Sea lamprey (*Petromyzon marinus*)	GCF_010993605.1	(Smith et al., 2018)	not present	XM_032950720.1	not present
Inshore hagfish (*Eptatretus burgeri*)	GCA_900186335.2	Riken Center for Development Biology	not present	ENSEBUT00000017110.1	ENSEBUT00000009389.1

The presence or absence of *slc26a12* was determined using synteny analysis. Genome databases of the vertebrate species listed in Table 1 were browsed using the Ensembl genome browser (https://www.ensembl.org) (Martin et al., 2023) or NCBI genome data viewer (https://www.ncbi.nlm.nih.gov/genome/gdv/) (Rangwala et al., 2021) and the presence of *slc26* gene around the *slc26a2* loci was manually examined.

### 2.2 Phylogenetic analysis and multiple amino‐acid sequence alignment

The amino acid sequences and accession numbers of the Slc26 family members of various vertebrate species listed in Figure 2 were collected from the GenBank/EMBL/DDBJ or Ensembl genome browsers. Some of these sequences were aligned with ClustalW software (Chenna et al., 2003) and ESPript (Robert & Gouet, 2014) was used to produce graphical display of the results.

For the phylogenetic analysis, all sequences listed in Figure 2 were aligned using ClustalW software, and the evolutionary history was inferred using the maximum likelihood method and the JTT matrix‐based model (Jones et al., 1992). The tree with the highest log‐likelihood (−102217.93) is shown. The percentages of trees in which the associated taxa were clustered together are shown below the branches. The initial tree(s) for the heuristic search were obtained automatically by applying the neighbor‐joining and BioNJ algorithms to a matrix of pairwise distances estimated using the JTT model and then selecting the topology with a superior log likelihood value. The tree was drawn to scale with branch lengths measured as the number of substitutions per site. The analysis included a total of 92 amino acid sequences. There were a total of 1240 positions in the final dataset. Evolutionary analyses were performed using MEGA11 software (Tamura et al., 2021).

### 2.3 Identification of the *slc26a12* pseudogene *slc26a12p*

This study uses the term “pseudogene” in a broad sense, including all cases in which the gene is predicted to not encode a full‐length solute carrier protein. When using “pseudogene” in a strict sense to include only cases in which it does not have any predicted function, the non‐functionality of pseudogenes can be difficult to define (Tutar, 2012). Therefore, in the broad sense here, the term includes the possibility that pseudogenes have functions other than that of solute carrier protein‐coding genes.

Dot plot analysis was performed to visualize the deletion in *slc26a12p*. To analyze *slc26a12p* in green sea turtles (*Chelonia mydas*), the ~30 kb genomic region of *slc26a2* and *slc26a12* in the common snapping turtle (*Chelydra serpentina*; ML689160.1:21238178–21,282,286) was compared with the corresponding ~60 kb region in the green sea turtle (NC_057854.1: 22082981–22,145,750). To analyze *slc26a12p* in owls, the ~20 kb genomic regions of *slc26a2* and *slc26a12* in Bengalese finches (*Lonchura striata*; NC_042579.1:17133908–17,165,633) were compared with the corresponding ~30 kb regions in burrowing owls (*Athene cunicularia*; NW_020799117.1:12171017–12,209,480). To analyze *slc26a12p* in penguins, the ~20 kb genomic regions of *slc26a2* and *slc26a12* in the Dalmatian pelican (*Pelecanus crispus*; NW_009101807.1:1–31,855) were compared with the corresponding ~20 kb regions in the emperor penguin (*Aptenodytes forsteri*; NW_008796044.1:6852725–6,885,690). To analyze *slc26a12p* in ducks, the ~30 kb genomic regions of *slc26a2* and *slc26a12* in chickens (*Gallus gallus*; NC_052544.1:7939223–7,974,822) were compared with the corresponding ~30 kb regions in the mallard (*Anas platyrhynchos*; NC_051785.1:20403555–20,446,036). Dot plot comparisons were performed using the European Molecular Biology Open Software Suite (EMBOSS) dot‐matcher program, with a window size of 20 and a threshold score of 70 (https://www.ebi.ac.uk/Tools/emboss/).

The pseudogenization of *slc26a12* was also analyzed by aligning the nucleotide sequences of regions corresponding to the protein‐coding regions of exons and identifying mutations, insertions, and deletions that generate stop codons or frameshifts. The orthologous sequences were identified by BLASTN search in the genome sequences of green sea turtle (*Chelonia mydas*; NC_057854.1), Eurasian eagle‐owl (*Bubo bubo*; ML981476.1), northern spotted owl (*Strix occidentalis caurina*; NIFN02011529.1), burrowing owl (*Athene cunicularia*; NW_020799117.1), little egret (*Egretta garzetta*; NW_009260839.1), emperor penguin (*Aptenodytes forsteri*; NW_008796044.1), rockhopper penguin (*Eudyptes chrysocome*; VULL01000090.1), yellow‐eyed penguin (*Megadyptes antipodes*; VULA01002606), Adelie pneguin (*Pygoscelis adeliae*; NW_008824283.1), Magellanic penguin (*Spheniscus magellanicus*; VULA01002606.1), mallard (*Anas platyrhynchos*; NC_051785.1), duck (*Anas platyrhynchos*; CM008551.1), Muscovy duck (*Cairina moschata*; QZEJ01003701.1), pink‐footed goose (*Anser brachyrhynchus*; NXHY01000155.1), swan goose (*Anser cygnoides*; NW_025927691.1), and black swan (*Cygnus atratus*; NC_066375.1). These sequences were aligned with *slc26a12* of common snapping turtle (*Chelydra serpentina*; JAHGAV010000001.1), Bengalese finch (*Lonchura striata*; NC_042579.1), Dalmatian pelican (*Pelecanus crispus*; NW_009101807.1), or chicken (*Gallus gallus*; NC_052544.1) using ClustalW software, and the mutations, insertions, and deletions that generate stop codons or frame shifts were manually identified using the translate tool in the Expert Protein Analysis System (ExPASy) web site (https://web.expasy.org/translate/).

### 2.4 Semiquantitative reverse transcription polymerase chain reaction (RT‐PCR)

Previously prepared total RNA from African clawed frogs (Motoshima et al., 2023, Tran et al., 2006) was used. First‐strand complementary DNA was synthesized from 5 μg of total RNA using the SuperScript IV First‐Strand Synthesis System (Thermo Fisher Scientific, Waltham, MA, USA) with oligo(dT) primers and analyzed by RT‐PCR, as described previously (Motoshima et al., 2023, Tran et al., 2006). cDNA was diluted eight‐fold with nuclease‐free water and used as a template for PCR with gene‐specific primers. The African clawed frog genome contains two sets of chromosomes, so there are two copies of *slc26a1*, *slc26a2*, and *slc26a12*, each on the L and S chromosomes. Primers were designed to amplify both copies, and the sequences and accession numbers are shown in Table 2. Each reaction mixture (final volume, 12.5 μL) consisted of 0.25 μL cDNA (template), primers (individual final concentration, 0.25 μM), and 6.25 μL GoTaq Green Master Mix (2×; Promega, Madison, WI, USA).

**TABLE 2:** Primers used for polymerase chain reaction amplification of *slc26a1*, *slc26a2*, and *slc26a12* in the African clawed frog.

Gene	Accession	Remarks	Direction	Sequence (5′–3′)
*slc26a1*	NM_001090973.1 (L) XM_041579722.1 (S)	RT‐PCR	Fw	agatctgccaactctgtggcg
			Rv	agagtcaattacagatgtgctgcagc
*slc26a2*	XM_018252193.2 (L) XM_018256127.2 (S)	RT‐PCR	Fw	actgataggtattgcattttccatg
			Rv	agtgcaaattggacagcttggt
*slc26a12*	XM_041586213.1 (L) XM_018256126.2 (S)	RT‐PCR	Fw	tctagctaaagtaactggcacag
			Rv	atctctttcagcacactaaggc
*actb.L*	NM_001088953.2	RT‐PCR	Fw	gacagtctgtgtgcgtccaa
			Rv	tgggcgacccacaatagatg

The PCR conditions were as follows: initial denaturation at 94°C for 2 min, 28 cycles (African clawed frog *slc26a1*, *slc26a2*, *slc26a12*, and *actb*) of 94°C for 15 s (denaturation), 54°C for 30 s (annealing), 72°C for 1 min (extension), and a final extension at 72°C for 7 min. After amplification, 3 μL of the PCR mixture was diluted and loaded onto a microchip electrophoresis system for DNA/RNA analysis (MCE‐202 MultiNA; Shimadzu, Kyoto, Japan) using a DNA‐2500 reagent kit (Shimadzu), according to the manufacturer's instructions. Electrophoresis results were analyzed using MultiNA Viewer software (Shimadzu).

## 3 RESULTS

### 3.1 Identification of *slc26a12* as a novel member of the *slc26* family

**FIGURE 1:**
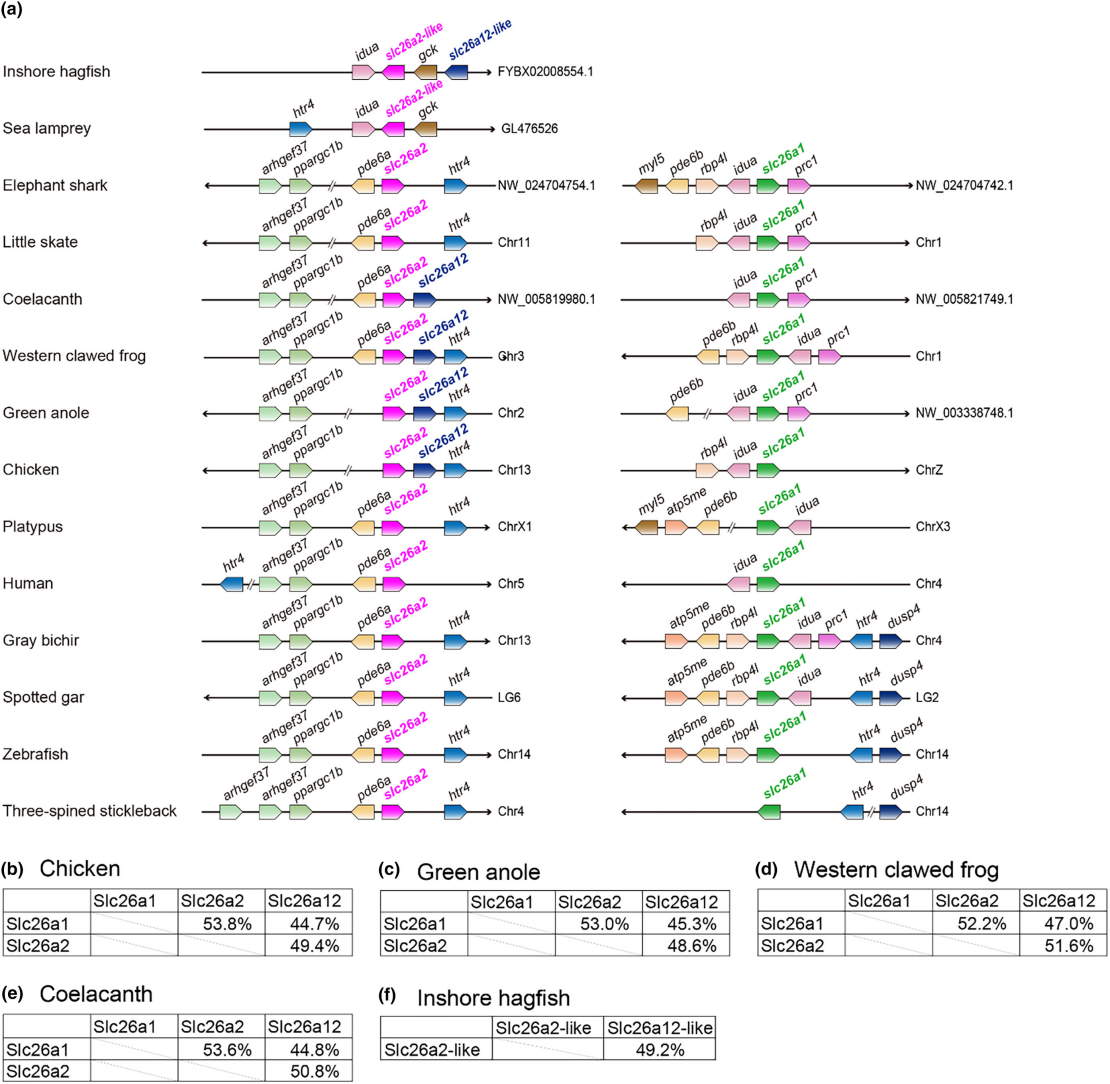
*slc26a12* in vertebrate species. (a) Synteny of *slc26a2*, *slc26a12* and *slc26a1* in vertebrates. Conserved synteny of *pde6a*/*pde6b*, *slc26a1*/*slc26a2*, and *htr4* in the genome database of indicated species are also shown. (b–f) Identity in amino‐acid sequences of Slc26a1, Slc26a2, and Slc26a12 in chicken (b), green anole (c), western clawed frog (d), and coelacanth (e). (f) Identity in amino‐acid sequences of Slc26a2‐like and Slc26a12‐like in inshore hagfish.

In the genome databases of coelacanths and nonmammalian tetrapod species, such as the western clawed frog, green anole, and chicken, an uncharacterized *slc26* gene, which we will name *slc26a12* in this study, was found downstream of the *slc26a2* locus (Figure 1a). The uncharacterized *slc26* gene was not found around *slc26a1* of all species examined and *slc26a2* of cartilaginous fishes, mammals, and ray‐finned fishes (Figure 1a).

**FIGURE 2:**
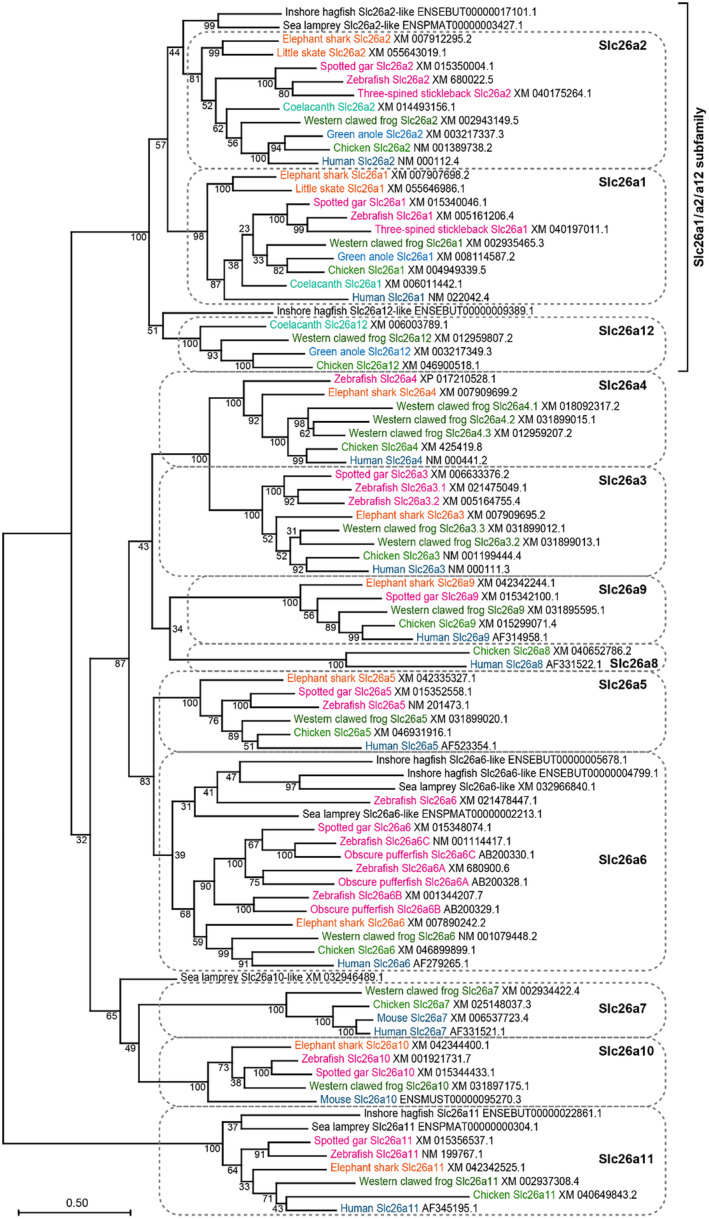
Phylogenetic analysis of Slc26a1, Slc26a2, and Slc26a12. The amino acid sequences of Slc26a1, Slc26a2, Slc26a12, and the other Slc26 members were aligned using ClustalW software. A phylogenetic tree was constructed using the maximum‐likelihood method and MEGA software. Numbers indicate bootstrap values, and the scale bar represents the genetic distance of amino acid substitutions per site. For the Slc26a1/a2/a12 subfamily, the amino acid sequences were obtained from 12 vertebrate species: inshore hagfish, sea lamprey, elephant shark, little skate, spotted gar, zebrafish, three‐spined stickleback, coelacanth, western clawed frog, green anole, chicken, and human. For the other Slc26 members, the amino acid sequences were obtained mainly from 8 vertebrate species: inshore hagfish, sea lamprey, elephant shark, spotted gar, zebrafish, western clawed frog, chicken, and human. The amino acid sequences of mouse Slc26a7 and Slc26a10 were included because human *slc26a10* is pseudogenized.

To analyze the evolutionary relationship of the new *slc26* gene, the predicted amino acid sequences were aligned with those of various Slc26s from tetrapods (human, chicken, green anole, and western clawed frog), lobe‐finned fish (coelacanth), ray‐finned fish (three‐spined stickleback and spotted gar), cartilaginous fish (little skate and elephant shark), and jawless fish (sea lamprey and inshore hagfish), and a phylogenetic tree was constructed (Figure 2). The new Slc26 of the coelacanth, western clawed frog, green anole, and chicken formed a single branch, which was independent of other Slc26 members, supported by a high bootstrap value (100%). Slc26a1 and Slc26a2 of cartilaginous fish, ray‐finned fish, coelacanths, and tetrapods also formed independent branches, supported by high bootstrap values (98 and 81%, respectively). Here, we refer to the new Slc26 as Slc26a12.

In the phylogenetic tree, three branches for Slc26a1, Slc26a2, and Slc26a12 in jawed vertebrates and the Slc26a2‐like and Slc26a12‐like in jawless fishes formed a single branch independent of the other Slc26 members, with a bootstrap value of 100. This result indicated that Slc26a1, Slc26a2, and Slc26a12 form a subfamily of Slc26.

Figure S1 shows multiple alignment of the amino acid sequences of Slc26a12. The amino acid sequences of Slc26a12 are 45%–47% and 49%–52% identical to those of Slc26a1 and Slc26a2, respectively, and this identity is slightly lower than the 52%–54% identity between Slc26a1 and Slc26a2 (Figure 1b–e). In inshore hagfish, the amino acid sequences of Slc26a12‐like are 49% identical to those of Slc26a2‐like (Figure 1f).

Recent analyses of the hagfish genome indicate that the 2R whole‐genome duplication in vertebrates occurred in the ancestral species of the jawed vertebrates (Yu et al., 2024). Synteny analyses of *slc26a1* and *slc26a2* showed the presence of phosphodiesterase 6B gene (*pde6b*) and *pde6a* in the loci of *slc26a1* and *slc26a2*, respectively. In addition, the 5‐hydroxytryptamine receptor 4 gene (*htr4*) was present in the loci of *slc26a1* and *slc26a2* in gray bichir and spotted gar and that of *slc26a2‐like* in sea lamprey (Figure 1a). These results suggest that *slc26a1* and *slc26a2* arose through the vertebrate 2R whole‐genome duplication.

### 3.2 *slc26a12* in lobe‐finned fishes and amphibians

**FIGURE 3:**
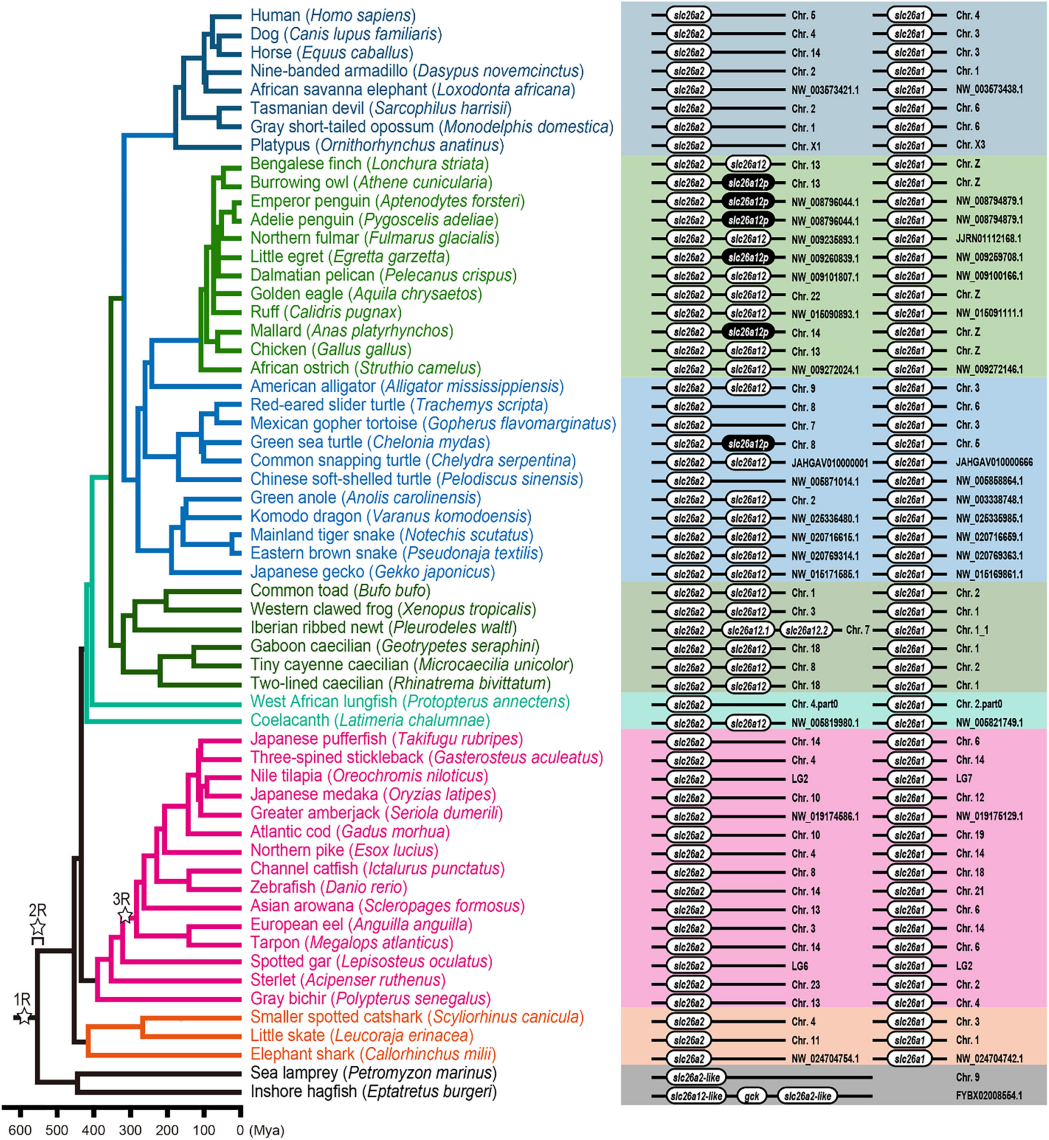
Presence of *slc26a12* as well as *slc26a2* and *slc26a1* in 58 vertebrate species. The accession number of each sequence is summarized in Table 1. Divergence times were retrieved from the TimeTree database (http://www.timetree.org/) (Kumar et al., 2017). *slc26a12p*, pseudogene of *slc26a12*. 1R–3R indicates the timing of whole‐genome duplications in early vertebrate evolution.

**FIGURE 4:**
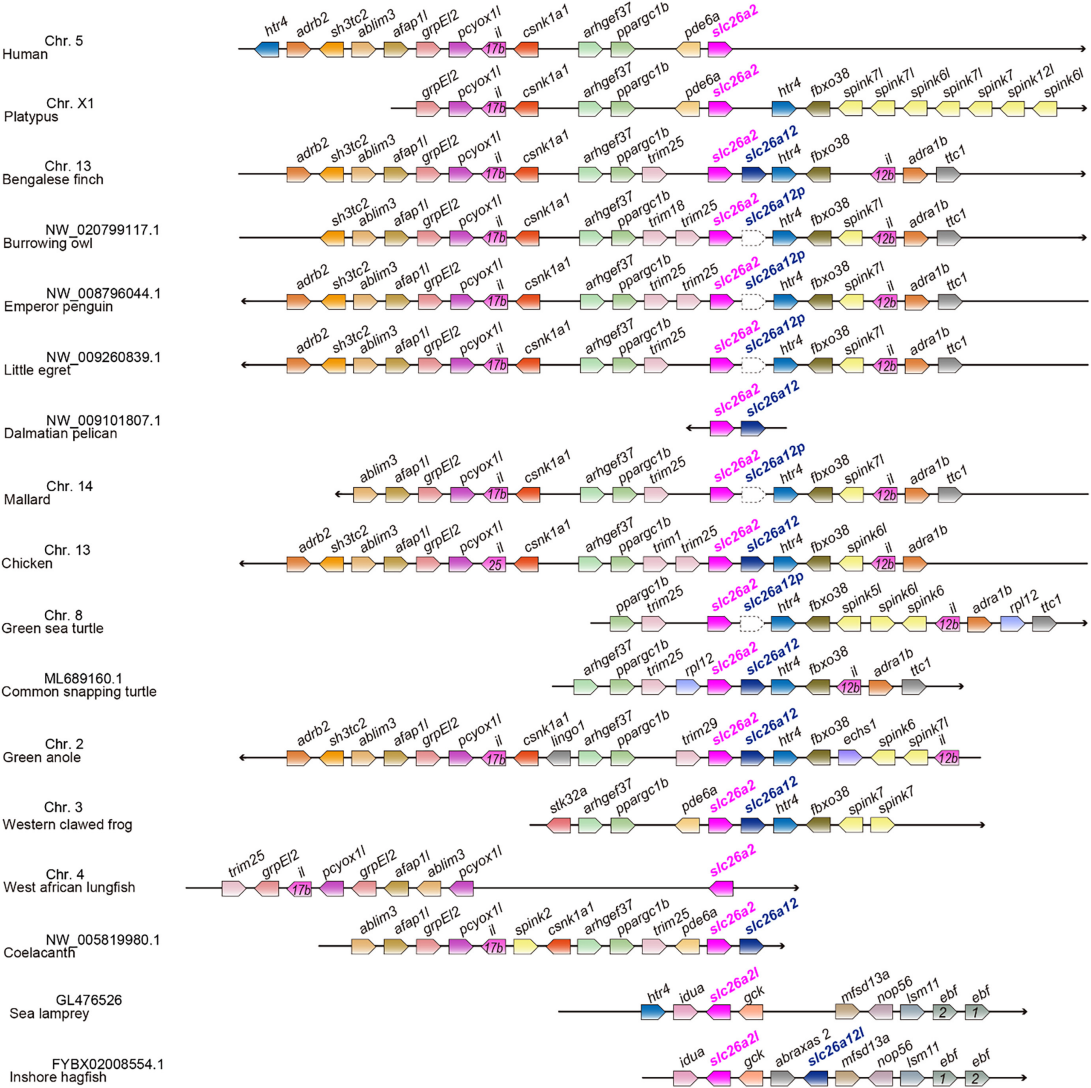
Synteny analyses of *slc26a12* in various vertebrate species.

Preliminary analyses suggest that there are species differences in the distribution of *slc26a12* in vertebrates. Therefore, we performed detailed synteny and BLAST analyses to analyze the presence or absence of *slc26a12* in different vertebrate lineages. The results are summarized in Figures 3 and 4 and detailed below.

In lobe‐finned fish, BLAST and synteny analyses showed that coelacanth has *slc26a12* whereas West African lungfish does not (Figures 3 and 4; Table 1). Both species harbor *slc26a1* and *slc26a2*.

In amphibians, all species of frogs, newts, and caecilians examined harbor *slc26a12*, and one each of *slc26a1* and *slc26a2*. The Iberian ribbed newt harbors two tandem paralogs of *slc26a12*, *slc26a12.1*, and *slc26a12.2* (Figures 3 and 4; Table 1).

### 3.3 *slc26a12* and *slc26a12p* in non‐avian reptiles

In non‐avian reptiles, all examined species of alligators, lizards, and snakes harbor *slc26a12*, *slc26a1*, and *slc26a2* (Figures 3 and 4; Table 1).

Among the turtles examined, only the common snapping turtle harbors *slc26a12* (Figures 3 and 4; Table 1). The green sea turtle has a pseudogene for *slc26a12*, *slc26a12p*, at the *slc26a12* locus. This study uses the term “pseudogene” in a broad sense, including all cases in which the gene is predicted not to encode a full‐length solute carrier protein as described in Materials and Methods. The red‐eared slider turtle, Mexican gopher tortoise, and Chinese soft‐shelled turtle lack *slc26a12*. All examined turtles have one each of *slc26a1* and *slc26a2*.

### 3.4 *slc26a12* and *slc26a12p* in birds

In the birds examined, most species, including finches, pelicans, eagles, chickens, and ostriches, harbor *slc26a12* (Figures 3 and 4; Table 1). The burrowing owl, emperor penguin, Adelie penguin, little egret, and mallard harbor *slc26a12p*. All birds examined have one each of *slc26a1* and *slc26a2*.

### 3.5 Lack of *slc26a12* in mammals, ray‐finned fishes, and cartilaginous fishes

All mammals, including monotremes, marsupials, Afrotheria, Xenarthra, and Boreoeutheria, lack *slc26a12* (Figures 3 and 4; Table 1). All ray‐finned fish, including bichers, gars, sturgeons, and teleosts lack *slc26a12* (Figures 3 and 4; Table 1). All cartilaginous fish, including elephant sharks, sharks, and rays lack *slc26a12* (Figures 3 and 4; Table 1). All of these species have one each of *slc26a1* and *slc26a2*.

### 3.6 *slc26a2‐like* and *slc26a12‐like* in jawless fishes

Phylogenetic analysis showed that the inshore hagfish and sea lamprey have two and one *slc26* genes that belong to the Slc26a1/a2/a12 subfamily, respectively (Figure 2). In this study, the inshore hagfish genes are tentatively referred to as *slc26a2‐like* and *slc26a12‐like*, and the sea lamprey gene is referred to as *slc26a2‐like*. Inshore hagfish Slc26a2‐like and sea lamprey Slc26a2‐like formed a single branch, indicating an orthologous relationship between the two genes. In the phylogenetic tree shown in Figure 2, Slc26a2‐like formed a branch with Slc26a2 of jawed vertebrates with a low bootstrap value (55%) and formed a branch with Slc26a1/Slc26a2 of jawed vertebrates with a low bootstrap value (60%). Therefore, the relationship between jawless fish Slc26a2‐like and jawed vertebrate Slc26a1/a2/a12 remains unclear. This result does not rule out the possibility that jawless fish Slc26a2‐like and jawed vertebrate Slc26a1/a2/a12 are orthologous.

In inshore hagfish, *slc26a2‐like* and *slc26a12‐like* genes are located close to each other on the same chromosome. In the phylogenetic tree, Slc26a12‐like formed a branch with Slc26a12 of jawed vertebrates with a low bootstrap value (54%). Therefore, the phylogenetic analysis did not clarify the relationship between jawless fish Slc26a2‐like and jawed vertebrate Slc26a1/a2/a12.

### 3.7 Tissue distribution of *slc26a12* in African clawed frogs

**FIGURE 5:**
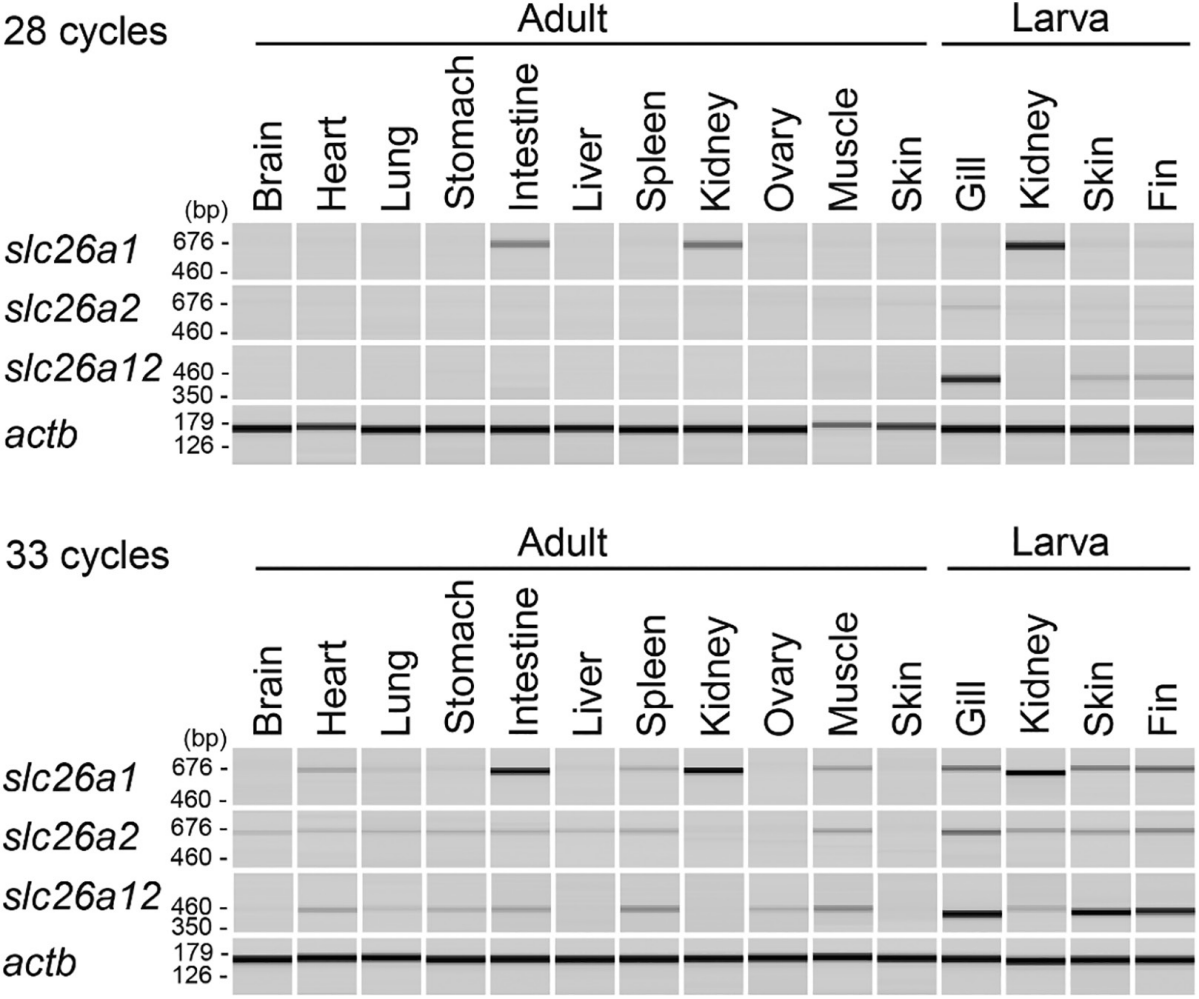
Tissue distribution of *slc26a12* in the African clawed frog. The results of semiquantitative RT‐PCR are shown as pseudo‐gel images of the PCR products using a microchip electrophoresis system. The single‐lane presentation is a feature of the electrophoresis system, and the reactions for each primer set were analyzed on the same electrophoresis run. Primers were designed to amplify both copies of *slc26a1*, *slc26a2*, and *slc26a12* on the L and S chromosomes. Primer sequences and accession numbers are shown in Table 2. Results for *slc26a1* and *slc26a2* are shown for comparison; *actb* (β‐actin gene) was used as an internal control.

The distribution of *slc26a1*, *slc26a2*, and *slc26a12* expression in African clawed frog tissues was analyzed using semiquantitative RT‐PCR (Figure 5). The African clawed frog is a tetraploid species, and each of *slc26a1*, *slc26a2*, and *slc26a12* was present in both of the L and S chromosomes. Therefore, we designed primers in the conserved regions and simultaneously amplified each gene in both chromosomes. The gene expression patterns were as follows: *slc26a1* was highly expressed in the intestine, kidney, and larval kidney and *slc26a2* in the larval gill and other various tissues; *slc26a12* was highly expressed in the larval gill, skin, and fin and at low levels in other tissues.

### 3.8 Pseudogenization of *slc26a12* in turtles and birds

**FIGURE 6:**
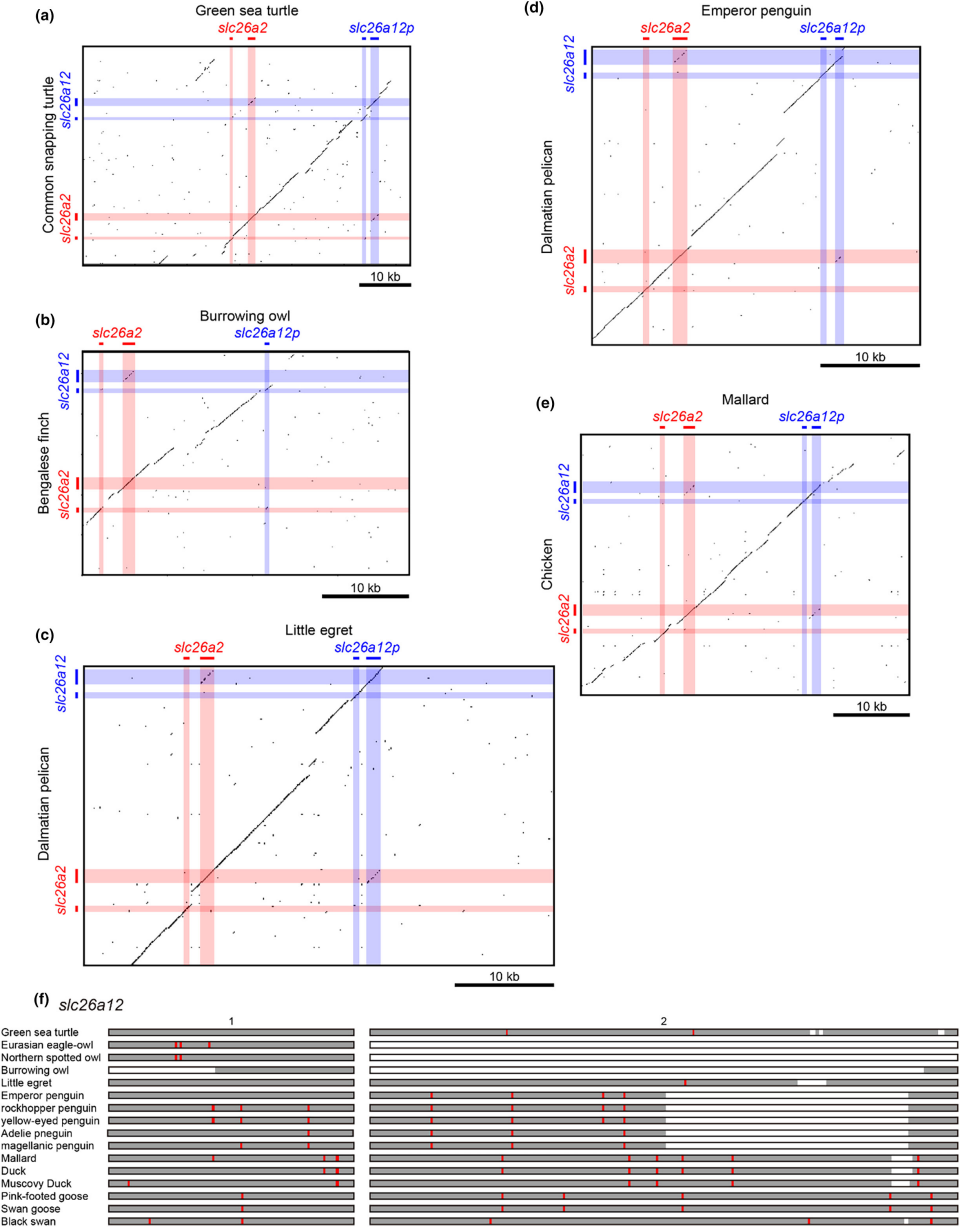
Pseudogenization of *slc26a12* in turtles and birds. Dot plot analyses of *slc26a2* and *slc26a12* compared to the corresponding genomic regions of green sea turtle (a), burrowing owl (b), little egret (c), emperor penguin (d), and mallard (e). The homologous regions were plotted using Dotmatcher (window size, 20; threshold, 70). (f) Deletion and nonsense mutations of *slc26a12p*. The schematic representations are based on the dot plot analyses above and a nucleotide sequence alignment in Figure S2. The open reading frames divided into two exons are represented by gray boxes. The deleted regions are indicated by open boxes. Nonsense mutations are indicated by red bars.

The presence of *slc26a12p* in turtles and birds was analyzed by dot plot analysis (Figures 6a–e) and by searching for point mutations, insertions, and deletions that produced in‐frame stop codons or frameshifts by aligning the nucleic acid sequences of regions orthologous to the exons of *slc26a12* (Figures 6f and S2). The genome sequence of the green sea turtle contains a pseudogene that was homologous to the entire region of *slc26a12* in the common snapping turtle and had nucleotide substitutions and deletions that generated in‐frame stop codons in exon 2 (Figure 6a, f, and S2).

The genome sequence of the burrowing owl contains a pseudogene homologous to the 5′ flanking region, exon 1, intron 1, and 3′ flanking region of *slc26a12* in the Bengalese finch (Figure 6b). Deletion of exon 2 in the pseudogene was also observed in other owls, such as the Eurasian eagle and northern spotted owls (Figures 6f and S2).

The little egret's genome sequence contains a pseudogene homologous to the entire region of *slc26a12* in Dalmatian pelicans (Figure 6c). The little egret *slc26a12p* has a mutation and a partial deletion that generated an in‐frame stop codon and frameshift, respectively, in exon 2 (Figure 6f and S2).

The genome sequence of the Emperor penguin contains a pseudogene that is homologous to the 5′ flanking region, exon 1, intron 1, 5′ and 3′ parts of exon 2, and 3′ flanking region of *slc26a12* in the Dalmatian pelican (Figure 6d). Deletion of part of exon 2 in the pseudogene was also observed in other owls, such as the rockhopper penguin, yellow‐eyed penguin, Adelie penguin, and Magellanic penguin (Figures 6f and S2). In addition, several point mutations that generated in‐frame stop codons were observed in both exons 1 and 2 of *slc26a12p* in penguins, and some of the in‐frame stop codons were conserved among *slc26a12p* in penguins (Figures 6f and S2).

The mallard genome sequence contains a pseudogene that was homologous to the entire region of *slc26a12* in chickens (Figure 6e). Several point mutations that generated stop codons were observed in both exons 1 and 2 of *slc26a12p* in ducks, such as mallard, duck, Muscovy duck, pink‐footed goose, swan goose, and black swan, and some of the in‐frame stop codons were conserved among *slc26a12p* in ducks (Figures 6f and S2).

## 4 DISCUSSION

In this study, we identified a new *slc26* gene that is located in tandem with *slc26a2* in several vertebrate lineages. Synteny and molecular phylogenetic analyses have confirmed the presence of orthologs of this gene in coelacanths, amphibians, reptiles, and birds (Figure 1). This result indicates that the gene is not a recent duplication in some specific species but was present at least before the divergence of tetrapods and coelacanths. Because this gene appeared in the very distant past, we propose to name it a new member of *slc26*, *slc26a12*. Molecular phylogenetic analysis indicated that *slc26a12* forms a subfamily along with *slc26a1* and *slc26a2*. The phylogenetic tree also indicated that the distance between Slc26a12 and Slc26a2 within the same species was greater than the distance between Slc26a2s in bony vertebrates and cartilaginous fishes. This result suggests two possibilities. First, gene duplication between *slc26a2* and *slc26a12* may have occurred before the separation of cartilaginous fish and bony vertebrates. Second, gene duplication between *slc26a2* and *slc26a12* may have occurred in the common ancestor of coelacanths and tetrapods after the separation of cartilaginous fish and bony vertebrates, and *slc26a12* may have evolved at a higher mutation rate than *slc26a2*.

A comparison of tissue distribution in African clawed frogs revealed that *slc26a12* had a different expression pattern than *slc26a1*, was relatively similar to that of *slc26a2*, and was most highly expressed in the larval gills, skin, and fin. This result suggests that *slc26a12* functions differently from *slc26a1* and may have a functional relationship with *slc26a2*. Its function and physiological role should be clarified in future studies.

*slc26a12* is not found in the genome databases of cartilaginous fish, ray‐finned fish, or mammals. In mammals, ancestral mammalian species probably lack *slc26a12*, as other tetrapods possess *slc26a12*. No pseudogenes for *slc26a12* were found in the mammalian genome database. Because some avian and reptilian lineages have lost or pseudogenized *slc26a12*, the deletion of *slc26a12* in mammals is considered to be in line with these lineages. In turtles, many species lack *slc26a12*, whereas the common snapping turtle has an intact *slc26a12* and the green sea turtle has an *slc26a12* pseudogene. The Chinese soft‐shelled turtle, the oldest divergent turtle species, lacks *slc26a12*, suggesting that gene deletion and pseudogenization occurred independently in multiple lineages of turtles. In birds, pseudogenes were found in owls, penguins, egrets, and ducks, and these pseudogenes share a common pattern of mutations and deletions in each lineage, but not between the lineages, suggesting that pseudogenes were generated independently in the ancestral species of owls, penguins, egrets, and ducks. Dot plot analyses indicated that *slc26a12p* in birds shows high homology with intact *slc26a12* in related species (Figure 6), suggesting that pseudogenization occurred relatively recently in the bird species. In general, pseudogenes are categorized into three groups: unprocessed duplicated, unprocessed unitary, and processed (Pink et al., 2011; Zhang et al., 2010). Unprocessed and processed pseudogenes are classified depending on whether they contain intron‐derived sequences; unprocessed pseudogenes are subcategorized as duplicated and unitary, depending on the presence of a functional parent gene. Dot plot analyses showed that *slc26a12p* are unprocessed because the homologous sequences were observed in both exon‐ and intron‐coding regions (Figure 6a–e). No functional parent gene was observed for *slc26a12p* in the avian and reptilian lineages and can thus be categorized as a unitary pseudogene. Most pseudogenes lose the ability to undergo transcription; however, there are examples of pseudogenes that are transcribed (Pink et al., 2011). A duplicated pseudogene can regulate the function of their parent genes via their transcripts (Lin et al., 2007; Scarola et al., 2015). Such mechanisms cannot be postulated in unitary pseudogenes because they lack a parent gene. To determine whether the *slc26a12p* has any function, further analysis will be required to evaluate in which species and organs it may be transcribed.

There are two possible explanations for why cartilaginous and ray‐finned fish lack *slc26a12*. The first scenario is that *slc26a12* is ancient in origin and has been present since before the divergence of the jawless and jawed species. In this case, the ancestors of cartilaginous and ray‐finned fish were secondarily deficient in *slc26a12*. If this scenario is correct, *slc26a12‐like* in hagfish may be an ortholog or closely related gene of *slc26a12*. The fact that hagfish *slc26a12‐like* is located near *slc26a2* on the same chromosome and the results of molecular phylogenetic analysis showed that *slc26a12‐like* and *slc26a12* are in a single branch, although the bootstrap value is low, does not exclude this possibility. In this case, lamprey would have lost its secondary *slc26a12‐like*. In a second scenario, *slc26a12* may be a paralog acquired by the common ancestors of coelacanths and tetrapods after their divergence from ray‐finned fishes. In this case, hagfish *slc26a12‐like* is a uniquely acquired paralog of their lineage. At this point, we find no evidence to confirm or refute either scenario. In both cases, *slc26a12* was present before the divergence of coelacanths and tetrapods; thus, it is a relatively ancient gene.

Since Slc26a12 has a primary structure ~50% identical to Slc26a1 and Slc26a2, it may have some function as an anion transporter, which should be clarified in the future study. The physiological significance of the presence of *slc26a12* in various lobe‐finned fishes and tetrapods and its absence in mammals, ray‐finned fishes, and cartilaginous fishes is unknown and remains to be elucidated. All jawed vertebrate species analyzed in this study have one *slc26a1* and one *slc26a2* without exception, and no species was found to be missing them or to have more than one paralog. This suggests that *slc26a1* and *slc26a2* are ubiquitously essential for the survival of jawed vertebrates. On the other hand, a difference in the presence or absence of the *slc26a12* was observed among the lineages, suggesting that the function of *slc26a12* is less important in some lineages, leading to its deletion or pseudogenization. The tissue distribution analysis in African clawed frog indicates that the *slc26a2* and *slc26a12* are expressed in relatively similar tissues. These results suggest that Slc26a12 may have redundant roles with Slc26a2. It is also possible that Slc26a2 compensates the function of Slc26a12 in lineages that do not have *slc26a12*. Several teleost groups display secondary loss of the stomach, and some genes involved in gastric acid secretion such as H^+^/K^+^‐ATPase (*atp4a* and *atp4b*), Cl^−^ channel‐transporter (*slc26a9*), and a regulatory subunit of the K^+^ channel (*kcne2*) have been lost or pseudogenized in agastric (stomachless) fishes (Kato et al., 2024; Castro et al., 2014). Slc26a9 has anion exchange, Cl^−^ channel, and Na^+^ coupled transporter activities and is involved in gastric and pulmonary functions (Chang et al., 2009); the loss or pseudogenization of *slc26a9* correlates with the secondary loss of the stomach in several lineages. In the case of *slc26a12*, the phenotype associated with the loss or pseudogenization of the *slc26a12* function is currently unknown.

The ancestors of turtles and birds have *slc26a12*, which has been pseudogenized in some of these lineages. However, the physiological significance that explains the pseudogenization of *slc26a12* in these species is also unclear. Turtles and birds include species with *slc26a12p* and those with intact *slc26a12*, both of the species are closely related, and the *slc26a12* pseudogenizations are relatively recent events. One possibility is that there is a large functional redundancy between *slc26a12* and *slc26a2* in these species and the difference between species in the degree of redundancy determines the tolerance for *slc26a12* pseudogenization.

### NEW & NOTEWORTHY

Slc26 belongs to an anion exchanger family consisting of 11 members in mammals. Based on the analysis of genome databases of various vertebrate species, we report Slc26a12 as a new member of the Slc26 family, which is widely present in coelacanths, amphibians, reptiles, and birds, but not in cartilaginous fishes, ray‐finned fishes, or mammals. This study provides an interesting sample for understanding how vertebrates have evolved a family of membrane transporters.

## AUTHOR CONTRIBUTIONS

A.N., K.T., and A.K. conceived and designed the research; A.N., K.T., O.C., and A.K. performed the experiments; A.N., K.T., O.C., and A.K. analyzed the data; A.N., K.T., O.C., and A.K. interpreted the results of the experiments; A.N., K.T., O.C., and A.K. prepared the figures; A.N., and A.K. drafted the manuscript; A.N., and A.K. edited and revised the manuscript; and A.N., K.T., O.C., and A.K. approved the final version of the manuscript.

## FUNDING INFORMATION

This study was supported by JSPS KAKENHI (Grant Numbers 21H02281 and 21 K14781).

## CONFLICT OF INTEREST STATEMENT

The authors declare no conflict of interest.

## ETHICS STATEMENT

All *Xenopus* care and animal protocols were conducted in accordance with the National Institutes of Health “Guide for the Care and Use of Laboratory Animals.” Frogs were housed and cared in accordance with a manual approved by the Institutional Animal Experiment Committee of the Tokyo Institute of Technology.

## Supporting information


Figure S1.

Figure S2.

## Data Availability

The data underlying this article are available in GenBank under the accession numbers indicated in Materials and Methods.
