# Supplementary material for: slc26a12—A novel member of the slc26 family, is located in tandem with slc26a2 in coelacanths, amphibians, reptiles, and birds
Source: Physiol Rep. 2024 Jun 3;12(11):e16089. doi: 10.14814/phy2.16089 (PMC11145369; doi:10.14814/phy2.16089)
Supplement: Supplementary file 1 — Figure S1. [file PHY2-12-e16089-s001.pdf]

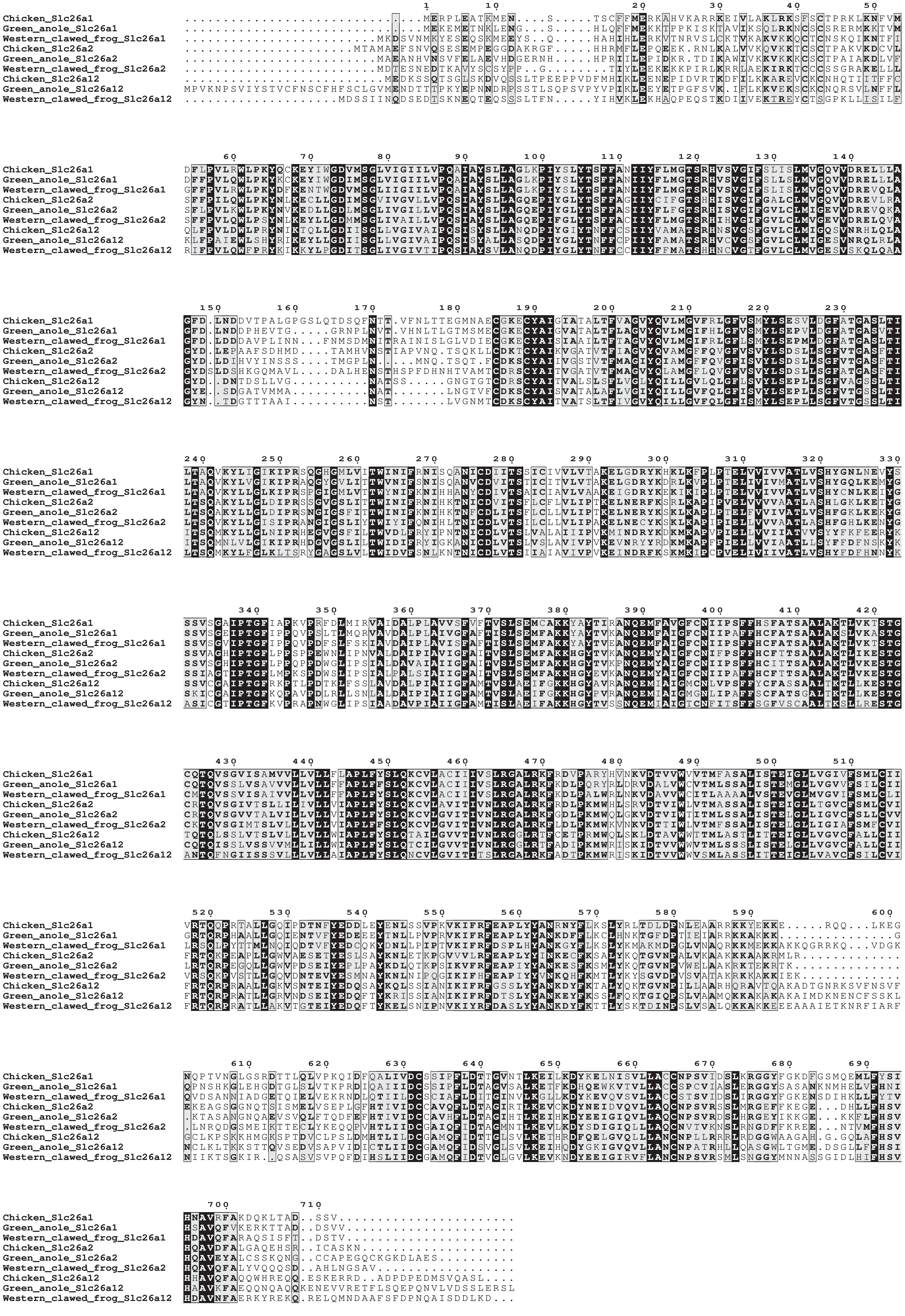

Figure S1. Multiple alignment of Slc26a1, Slc26a2, and Slc26a12 in chicken, green anole, and western clawed frog.
