# Supplementary material for: slc26a12—A novel member of the slc26 family, is located in tandem with slc26a2 in coelacanths, amphibians, reptiles, and birds
Source: Physiol Rep. 2024 Jun 3;12(11):e16089. doi: 10.14814/phy2.16089 (PMC11145369; doi:10.14814/phy2.16089)
Supplement: Supplementary file 2 — Figure S2. [file PHY2-12-e16089-s002.pdf]

A Exon1

Common snapping turtle *slc26a12*	ATGGAGACTGCCACAACACGGGAGTTTGAGCAGAGCATAGAAAAACTGAG
Green sea turtle *slc26a12p*	ATGGAGACTGCCACAAAACAGAAGTTTGAGCAGAGCATAGAAACACTGAG
Bengalese finch *slc26a12*	ATGGAGGACACGTCCGCGGAGGAGCCGGCGCCCAGCAAAGCCACCCTGGG
Eurasian eagle owl *slc26a12p*	ATGGAAGACACATCAAGCCAGAAGTCAGGACAGAGCAACGACATACTGAG
Northern spotted owl *slc26a12p*	ATGGAAGACACATCAAGCCAGAAGTCAGGACAGAGCAATGACATACTGAG
Burrowing owl *slc26a12p*	--
Dalmatian pelican *slc26a12*	ATGGAGGACACATCAAGCCAGAAGTCAGGACAGAGCAAAGACGCACTGGG
Little egret *slc26a12p*	ATGGAGGACACATCAAGCCAGAAGTCAGGACAGAGCAAAGATGCATGGAG
Emperor penguin *slc26a12p*	ATGGAGGACACATCAAGCCAGAAGTCAGGACAGAGCAAAGACGCACTGGG
Rockhopper penguin *slc26a12p*	ATGGAGGACACATCAAGCCAGAAGTCAGGACAGAGCAAAGACGCGCTGGG
Yellow-eyed penguin *slc26a12p*	ATGGAGGACACATCAAGCCAGAAGTCAGGACAGAGCAAAGACGCGCTGGG
Adelie penguin *slc26a12p*	ATGGAGGACACATCAAGCCAGAAGTCAGGACAGAGCAAAGACGCACTGGG
Magellanic penguin *slc26a12p*	ATGGAGGACACATCAAGCCAGAAGTCAGGACAGAGCAAAGACGCGCTGGG
Chicken *slc26a12*	ATGGAGGACAAGTCAAGCCAGACGTCAGGACTGAGCAAAGATGTACAGAG
Mallard *slc26a12p*	ATGGAAGGCACACTGAGCCAGATATCAGGACAGAGCAAAGATGTACCAAG
Duck *slc26a12p*	ATGGAAGGCACACTGAGCCAGATATCAGGACAGAGCAAAGATGTACCAAG
Muscovy duck *slc26a12p*	ATGGAAGGCACACTGAGCCAGATATCAGGACAGAGCAAAGATGTACGAG-
Pink-footed goose *slc26a12p*	ATGGAAGGCATGCTGAGCCAGATGTCAGGACAGAGCAAAGATGTACTGAG
Swan goose *slc26a12p*	ATGGAAGGCATGCTGAGCCAGATGTCAGGACAGAGCAAAGATGTACTGAG
Black swan *slc26a12p*	ATGGAAGGCACACCGAGCCAGACGACAGGACAGAGCAAAGATGTACTGAG

Common snapping turtle *slc26a12*	CTCCTCTGCGCCACAAGATTCATCTTTTCGTCACATACACATCAAGTTAG
Green sea turtle *slc26a12p*	CTCCTCTGCACCACAAGATTCATCTTTTTATCACATACACATCAAATTAG
Bengalese finch *slc26a12*	CTCGCCCCGGCCTCACGAGGCTCCCGCCACCTTTG------TCAGCCTGG
Eurasian eagle owl *slc26a12p*	CTCCCTAACACCAGAAGAAAGTCCTGCCAATTTTATACATGTCAGACCAG
Northern spotted owl *slc26a12p*	CTCCCTAACACCAGAAGAGAGTCCTGCCAATTTTATACATGTCAGATCAG
Burrowing owl *slc26a12p*	--
Dalmatian pelican *slc26a12*	CTCCCTAACACCAGAAGAGAGTCCTGCCAATTTTATACATGTCAAATTGG
Little egret *slc26a12p*	CTCCCTAACGCCAGAAGAGAGTCCTGCTGATTTTATATGTGTCAGATTGG
Emperor penguin *slc26a12p*	CTCCCTAACACCAGAAGAGAGTCCTGCCAATTTTATACATGTCAAATTGG
Rockhopper penguin *slc26a12p*	CTCCCTAACACCAGAAGAGAGTCCTGCCAATTTTATACATGTCAAATTGG
Yellow-eyed penguin *slc26a12p*	CTCCCTAACACCAGAAGAGAGTCCTGCCAATTTTATACATGTCAAATTGG
Adelie penguin *slc26a12p*	CTCCCTAACACCAGAAGAGAGTCCTGCCAATTTTATACATGCCAAATTGG
Magellanic penguin *slc26a12p*	TTCCCTAACACCAGAAGAGAGTCCTGCTAATTTTATACATGTCAAATTGG
Chicken *slc26a12*	CTCCCTAACACCAGAAGAGCCTCCTGTCGACTTTATGCACATCAAGTTGG
Mallard *slc26a12p*	CTCCCTAACCCCAGAAGAGCCTCCTATCAATTTTACACATGTCAGGTTGG
Duck *slc26a12p*	CTCCCTAACCCCAGAAGAGCCTCCTATCAATTTTACACATGTCAGGTTGG
Muscovy duck *slc26a12p*	CTCCCTAACCCCAGAAGAGCCTCCTATCAATTTTACACGTGTCAGGTTGG
Pink-footed goose *slc26a12p*	CTCCCTAACCCCAGAAGAGCCTCCTGTCAATTTTATACGCGTCAGGTTGG
Swan goose *slc26a12p*	CTCCCTAACCCCAGAAGAGCCTCCTGTCAATTTTATACGCGTCAGGTTGG
Black swan *slc26a12p*	CTCCCTAACCCCAGAAGAGCCTCGTGACAATTTTATATGCATCAGGTTAG

Common snapping turtle *slc26a12*	AGGAGCATGAGCCCACAAGTCTTAGCA----------CCAAGGAGCTCAT
Green sea turtle *slc26a12p*	AGGAGCATGAGCCCACAAGTCTTAGCA----------CCAAGGAGCTCAT
Bengalese finch *slc26a12*	AGGAGCAGGAGCCCGCGGCCTGCAGCG----------CCGGGGAGCTGAT
Eurasian eagle owl *slc26a12p*	AGGAGTATGAGCCCGCAGACTTCAGCA----------CCAAGGATCTCAT
Northern spotted owl *slc26a12p*	AGGAGTATGACCCCGCAGACTTCAGCA----------CCAAGGATCTCAT
Burrowing owl *slc26a12p*	--
Dalmatian pelican *slc26a12*	AGGAGTATGAGCCTGCAGACTTCAGCA----------CCAAGGAACTCAT
Little egret *slc26a12p*	AGGAGTATGAGCCCGCAGACTTTGGCA----------CCAAGGAACTCAT
Emperor penguin *slc26a12p*	AGGACTATGAGCCTGCAAACTTCAGCA----------CCAAGGAACTCAT
Rockhopper penguin *slc26a12p*	AGGACTATGAGCCTGCAGACTTCAGCAGGCTGAAGGAACAAGGAACTCAT
Yellow-eyed penguin *slc26a12p*	AGGACTATGAGCCTGCAGACTTCAGCAGGCTGCAGGAACAAGGAACTCAT
Adelie penguin *slc26a12p*	AGGACTATGAGCCTGCGGACTTCAGCA----------CCAAGGAACTCAT
Magellanic penguin *slc26a12p*	AGGACTATGAGCCTGCAGACTTCAGCA----------CCAAGGAACTCAT
Chicken *slc26a12*	AGGAGAATGAGCCCATAGACGTCAGGA----------CCAAGGATTTCAT
Mallard *slc26a12p*	AGGAGAATGAGCCCACAGACTTCAGCA----------CCAAGGATTTCAT
Duck *slc26a12p*	AGGAGAATGAGCCCACAGACTTCAGCA----------CCAAGGATTTCAT
Muscovy duck *slc26a12p*	AGGAGAATGAGCCCACAGACTTCAGCA----------CCAAGGATTTCAT
Pink-footed goose *slc26a12p*	AGGAGAATGAGCCCACAGACTTCAGCA----------CCAAGGATTTCAT
Swan goose *slc26a12p*	AGGAGAATGAGCCCACAGACTTCAGCA----------CCAAGGATTTCAT
Black swan *slc26a12p*	AGTAGAACGAGCCCACAGACTTCAGCA----------CCAAGGATTTCAT

Common snapping turtle *slc26a12*	CCTGAAGAAAGCCAAAGAAGCTTGCAAGTGGAATCACCAAATTGTTATCA
Green sea turtle *slc26a12p*	CCTGAAGAAAGCCAAAGAAGCTTGCAAGTGGAATCGCCAAATTGTCATCA
Bengalese finch *slc26a12*	CCTGCAGGAGGCCAGGGCGGCGTGCGGGCGCCGCGGGCCG------GGCG
Eurasian eagle owl *slc26a12p*	CCTAAAGAAAGCCAGAGAGTTCTGCACATGCAGTCATCGAACCATCCTCA
Northern spotted owl *slc26a12p*	CCTAAAGAAAGCCAGAGAGTTCTGCACATGCAGTCATCGAACCATCCTCA
Burrowing owl *slc26a12p*	--
Dalmatian pelican *slc26a12*	CCTAAAGAAAGCCAGAGAGGTCTGCACATGCAGTCATCAAACTATCATCC
Little egret *slc26a12p*	CCTAAAGAAAGCCAGAGAGGTCTGCACATGCAATCATCAAACCATCATCA
Emperor penguin *slc26a12p*	TCTAAAGAAAGCCAGAGAGGTCTGCACATGCAATCATCAAACCATCATCA
Rockhopper penguin *slc26a12p*	TCTAAAGAAAGCCAGAGAGGTCTGCACATGCAATCATCAAACCATCATCA
Yellow-eyed penguin *slc26a12p*	TCTAAAGAAAGCCAGAGAGGTCTGCACATGCACTCATCAAACCATCATCA
Adelie penguin *slc26a12p*	TCTAAAGAAAGCCAGAGAGGTCTGCACATGCAATCATCAAACCATCATCA
Magellanic penguin *slc26a12p*	TCTAAAGAAAGCCAGAGAGGTCTGCACATGCAATCATCAAACCATCATCA
Chicken *slc26a12*	CCTGAAAAAAGCCAGAGAGGTCTGCAAATGCAACCACCAAACCATCATCA
Mallard *slc26a12p*	CCTGAAGAAAGCCAGAGAGGTCTGCAAATGCA---ATCATCAAACCATCA
Duck *slc26a12p*	CCTGAAGAAAGCCAGAGAGGTCTGCAAATGCAATCATCAAACCATCATCA
Muscovy duck *slc26a12p*	CCTGTAGAAACCCAGAAAGGTCTGCAATC------ATCAAACCATCATCA
Pink-footed goose *slc26a12p*	CCTGAAGAAAGCCAGAGAGGTCTGCAAATGCAATCATCAAACCATCCTCA
Swan goose *slc26a12p*	CCTGAAGAAAGCCAGAGAGGTCTGCAAATGCAATCATCAAACCATCCTCA
Black swan *slc26a12p*	CCTGAAGAAAGCCAGAGAGGTCTGCAAATGCAATCATCAAACCATCCTCA

Common snapping turtle *slc26a12*	CTTTCTTCCTCAAGCTGCTCCCAGTGCTGGAGTGGCTTCCCCGATACAGA
Green sea turtle *slc26a12p*	CTTTCTTCCTCAAGCTGCTCCCAGTGCTGGAATGGCTTCCCCGACACAGA
Bengalese finch *slc26a12*	CGTGGCTGCGCCGCCTGCTCCCGGTGCTGGTGTGGCTGCCCCGCTACAGC
Eurasian eagle owl *slc26a12p*	CTTTCTTCTGTCAGCTGTTCCCAGTGCTGGACTGGCTTCCCTGTTACAAT
Northern spotted owl *slc26a12p*	CCTTCTTCTGTCGGCTGTTCCCAGTGCTGGACTGGCTTCCCTGTTACAAT
Burrowing owl *slc26a12p*	--TTCAAGAGTAGGGTCGACAGAGCGCTGAGGG-----------------
Dalmatian pelican *slc26a12*	CCTTCTTCTGCCAGTTGTTCCCAGTGCTGGACTGGCTTCCCCATTACAAT
Little egret *slc26a12p*	CCTCCTTCTTTCAGCTATTCCCAGTGCTGGACTGGCTTCCCCGTTACAAC
Emperor penguin *slc26a12p*	CCACCTTCTGTCGGCTGTTCTCAGTGCTGGACTGGCTTCCCCGTTACAAC
Rockhopper penguin *slc26a12p*	CCACCTTCTGTCGGCTGTTCTCAGTGCTGGACTGGCTTCCACGTTACAAC
Yellow-eyed penguin *slc26a12p*	CCACCTTCTGTCGGCTGTTCTCAGTGCTGGACTGGCTTCCACGTTACAAC
Adelie penguin *slc26a12p*	CCACCTTCTGTCGGCTGTTCTCAGTGCTGGACTGGCTTTCCCATTACAAC
Magellanic penguin *slc26a12p*	CCACCTTCTGTCGGCTGTTCTCAGTGCTGGACTGGCTTCCCCATTACAAC
Chicken *slc26a12*	CCTTCTTCTGCCAGCTGTTCCCGGTGCTAGACTGGCTTCCCCGTTACAAC
Mallard *slc26a12p*	TCACCTTCTGTCGGCTGTTCCCAGTGCTGGACTGGCTGCCCCGGTACGAT
Duck *slc26a12p*	CCTTCTTCTGTCGGCTGTTCCCAGTGCTGGACTGGCTGCCCCGGTACGAT
Muscovy duck *slc26a12p*	CCTTCTTCTGTTGGCTGTTCCCAGTGCTGGACTGGCTGCCCCGGTACGAT
Pink-footed goose *slc26a12p*	CCTTCTTCTGTCGGCTGTTCCCAGTGCTGAACTGGCTGCCCCGGTACGAT
Swan goose *slc26a12p*	CCTTCTTCTGTCGGCTGTTCCCAGTGCTGAACTGGCTGCCCCGGTATGAT
Black swan *slc26a12p*	CCTTCTCCTGTCGGCTGTTCTCAGTGCTGGACTGGCTGCCCCGGTACGAT

Common snapping turtle *slc26a12*	ATCAAGGAACAGTTGCTTGGGGATGTCATCTCTGGATTACTGGTCG---G
Green sea turtle *slc26a12p*	ATCAAGGAACAGTTGCTTGGGGATGTCATCTCTGGATTACTGGTTG---G
Bengalese finch *slc26a12*	CCCCGCACCCAGCTGCTGGGGGACGTGGTCTCGGGGCTCCTGGTGG---G
Eurasian eagle owl *slc26a12p*	GTCAAGAC-CAGTTGCTGGGGGATGTCATATCTGGGCTGCTGGTGG---G
Northern spotted owl *slc26a12p*	GTCAAGACGCGGTTGCTTGGGGATGTCATATCTGGGCTGCTGGCAG---G
Burrowing owl *slc26a12p*	----ATATGGTGTAGTTGGGAACTGTCAGTGTGAGGTTAATGGTTG----
Dalmatian pelican *slc26a12*	GTCAAGACGCAGTTGCTTGGGGATGTCATATCTGGGCTCCTGGTGG---G
Little egret *slc26a12p*	GTCAAGATGCAGCTGCTTGGGGATGTCATATCTGGGCTCCTGGTGG---G
Emperor penguin *slc26a12p*	GTCAAGACGCAGTTGCTTGGGGATGTCATATCTGGGCTCCTGGTGG---G
Rockhopper penguin *slc26a12p*	GTCAAGATGCAGTTGCTTGGGGATGTCATATCTGGGCTCCTGG------G
Yellow-eyed penguin *slc26a12p*	GTCAAGATGCAGTTGCTTGGGGATGTCATGTCTGGGCTCCTGG------G
Adelie penguin *slc26a12p*	GTCAAGATGCAGTTGCTTGGGGATGTCATATCTGGGCTCCTGGTGG---G
Magellanic penguin *slc26a12p*	GTCAAGATGCAGTTGCTTGGGGATGTCATATCTGGGCTCCTGGTGG---G
Chicken *slc26a12*	ATCAAGACGCAGCTGCTTGGGGACATCATATCTGGGCTCCTGGTGG---G
Mallard *slc26a12p*	GTCAGGACTCAGTTCCTTGGG-----CGTACCTGGGCTCCTGGTGG---G
Duck *slc26a12p*	GTCAGGACTCAGTTCCTTGGGGATGGCGTACCTGGGCTCCTGGTGG---G
Muscovy duck *slc26a12p*	GTCAGGACTCAGTTCCTTGGGGATGGCATATCTGGGCTCCTGGTGG---G
Pink-footed goose *slc26a12p*	GTCAGGACGCAGTTACTTGGGGATGTCGTATCTGGCCTCCTGGAGGCCAG
Swan goose *slc26a12p*	GTCAGGACACAGTTACTTGGGGATGTCGTATCTGGCCTCCTGGAGGCCAG
Black swan *slc26a12p*	GTCAGGACACAGTTACTTGTGGGTGTCGTATCTGGGCTCCTGGTGG---G

Common snapping turtle *slc26a12*	GATAGTTGCCATTCCACAGTCAATCTCCTACTCCCTCTTAGCAAGCCAGG
Green sea turtle *slc26a12p*	GATAGTTGCCATCCCACAATCAATCTCCTACTCCCCCTTATCAAGCCAGG
Bengalese finch *slc26a12*	CGTGGTGGCCATCCCCCAGTCCATCTCCTACTCGCTGCTGGCCAACCAGG
Eurasian eagle owl *slc26a12p*	GATAGCTGCCATCCCTCAGTCCATCTCCTACTCCCTCTTATCCAACCAGG
Northern spotted owl *slc26a12p*	GATAGTTGCCATCCCTCAGTCCATCTCCTACTCCCTCTTATCCAACATGG
Burrowing owl *slc26a12p*	----------------GACTGGATGATCTTCAAGGTCTTTGCCAACTGAG
Dalmatian pelican *slc26a12*	GATAGTTGCCATCCCTCAGTCCATCTCCTACTCCATCTTAGCCAACCAGG
Little egret *slc26a12p*	GATAGTTGCCATCCCTCAGTCCATCTCCTACTCTGTCTTAGCCAACCAGG
Emperor penguin *slc26a12p*	GATAGTTGCCATCCCTCAGTCCATCTCCTACTCCCTCTTAGCCAACCAGG
Rockhopper penguin *slc26a12p*	GATAGTTGCCATCCCTCAGTCCATCTCCTACTCCCTCTTAGCCAACCAGG
Yellow-eyed penguin *slc26a12p*	GATAGTTGCCATCCCTCAATCCGTCTCCTACTCCCTCTTAGCCAACCAGG
Adelie penguin *slc26a12p*	GATAGTTGCCATCCCTCAGTCTGTCTCCTACTCCCTCTTAGCCAACCAGG
Magellanic penguin *slc26a12p*	GATAGTTGCCATCCCTCAGTCCGTCTCCTACTCCCTCTTAGCCAACCAGG
Chicken *slc26a12*	GATAGTCGCCATCCCTCAGTCCATCTCGTACTCCCTGTTAGCCAACCAGG
Mallard *slc26a12p*	GACAGTTGCCATCCCTCAGTCCATCTCGCACTCGCTGCTAGCCAGCCAGG
Duck *slc26a12p*	GACAGTTGCCATCCCTCAGTCCATCTCGTACTCGCTGCTAGCCAGCCAGG
Muscovy duck *slc26a12p*	GACGGTTGCCATCCCTCAGTCCATCTCGTACTCGCTGCTAGCCAGCCAGG
Pink-footed goose *slc26a12p*	GATAGTTGCCATCCCTCAGTCCGTCTCGTACTCCCTGCTAGCCAGCTAGG
Swan goose *slc26a12p*	GATAGTTGCCATCCCTCAGTCCGTCTCGTACTCCCTGCTAGCCAGCTAGG
Black swan *slc26a12p*	GATAGCTGCCATCCCTCAGTCCATCTCGTACTCCCTGCTAGCCAGCTAGG

Common snapping turtle *slc26a12*	ACCCCATCTACGGACTCTACACCAACTTCTTCTGCTCCGTCATCTACTTT
Green sea turtle *slc26a12p*	ACCCCATCTATGGACTCTACACCAACTTCTTCTGCTCCATCATCTACTTT
Bengalese finch *slc26a12*	ACCCCATCTACGGCATCTACACCAACTTCTTCTGCAACATCATCTACGCG
Eurasian eagle owl *slc26a12p*	ATCCCATCTGTGGAATCTACACCAGCTTCTTCTGCAATATCATCTACGTC
Northern spotted owl *slc26a12p*	ATCACATCTATGGAATCTACACCAGCTTCCTCTGCAATATCATCTACGTC
Burrowing owl *slc26a12p*	AAGATTCTGTG--AATCTACACCAGCTTCTTCTGCAATATCATCTACGTC
Dalmatian pelican *slc26a12*	ATCCCATCTACGGAATCTACACCAACTTCTTCTGCAATATCATCTACGTT
Little egret *slc26a12p*	ATCCCATCTATGGAATCTACACCAACTTCTTCTGCAATATCATCTATGTT
Emperor penguin *slc26a12p*	ATCCCATCTACGGAATCTACACCAACTTCTTCTGCGATATCATCTACATT
Rockhopper penguin *slc26a12p*	A-CCCATCTATGGAATCTACACCAACTTCTTCTGCAATATCATCTACATT
Yellow-eyed penguin *slc26a12p*	A-CCCATCTATGGAATCTACACCAACTTCTTCTGCAATATCATCTACATT
Adelie penguin *slc26a12p*	A-CCCATCTATGGAATCTACACCAACTTCTTCTGCAATATCATCTACATT
Magellanic penguin *slc26a12p*	A-CCCATCTATGGAATCTACACCAACTTCTTCTGCAATATCATCTACATT
Chicken *slc26a12*	ATCCCATCTATGGAATCTACACAAACTTCTTCTGCAGTATCATCTACGTC
Mallard *slc26a12p*	ATCTCATCTACGGAATCTACACCAACTTCTTCTGCAATATCATCTATGTG
Duck *slc26a12p*	ATCTCATCTACGGAATCTACACCAACTTCTTCTGCAATATCATCTATGTG
Muscovy duck *slc26a12p*	ATCTCATCTACGGAATCTACACCAACTTCTTCTGCAATATCATCTATGTG
Pink-footed goose *slc26a12p*	ATCCCATCTACGGAATCTACACCAACTTCT---GCAATATCATCTACGTG
Swan goose *slc26a12p*	ATCCCATCTACGGAATCTACACCAACTTCT---GCAATATCATCTACGTG
Black swan *slc26a12p*	ATCCCATCTACGGAATCTACACCAACTTCTTCTGCAATATCATCTACGTG

Common snapping turtle *slc26a12*	GTCACGGCCACCTCGCGCCATAATGCTGTCGGCTCCTTCGGTGTCCTGTG
Green sea turtle *slc26a12p*	GTCATGGCCACCTCGCGCCATAATGCTGTCGGGTCCTTTGGTGTCCTGTG
Bengalese finch *slc26a12*	GCCGCGGCCACGTCGCGCCACGCCAGCGTGGGCTCCTTTGGGGTGCTGTG
Eurasian eagle owl *slc26a12p*	GCTATAGCCACGTCACAGCATAACTTCATGGGCTGCTTCGGTGTCCTGTG
Northern spotted owl *slc26a12p*	GCTATAGCCACATCACGGCATAACTTCATGGGCTCCTTCGGCGTCCTGTG
Burrowing owl *slc26a12p*	GCTATAGCCATGTCACAGCATAGCCTCCTGGGCTCCTTCGGCATCCTGTG
Dalmatian pelican *slc26a12*	GCTATGGCCACTTCACGCCATAACTTCGTGGGCTCCTTCGGCGTCCTGTG
Little egret *slc26a12p*	GCTATGGCCACTTCACGCCATAACTTTGTGGGCTCCTTTGGCGTGCTGTG
Emperor penguin *slc26a12p*	GCTATGGCCACTTTGCGCCGTAACTTCATGGGCTCCTTCGGTGTCCCATG
Rockhopper penguin *slc26a12p*	GCTATGGCCACTTTGCGCCGTAACTTCATGGGCTCCTTCGGTGTCCCGTG
Yellow-eyed penguin *slc26a12p*	GCTATGGCCACTTTGCGCCGTAACTTCATGGGCTCCTTTGGTGTCCCGTG
Adelie penguin *slc26a12p*	GCTATGGCCACTTTGCGCCGTAACTTCATGGGCTCCTTCGGTGTCCCATG
Magellanic penguin *slc26a12p*	GCTATGGCCACTTTGCGCCGTAACTTCATGGGCTCCTTCGGTGTCCCGTG
Chicken *slc26a12*	GCCATGGCCACTTCACGCCACAACTCTGTGGGATCCTTCGGTGTCCTTTG
Mallard *slc26a12p*	GCCATGGCCACTTCACGACACAACTTCATTGGATCCTTCGGAGTCCTTTG
Duck *slc26a12p*	GCCATGGCCACTTCACGACACAACTTCATTGGATCCTTCGGAGTCCTTTG
Muscovy duck *slc26a12p*	GCCATGGCCACTTCACGCCACAACTTTGTTGGATCCTTTGGGGTCCTTTG
Pink-footed goose *slc26a12p*	GCCATGGCTACTTCACACCACAACTTCGCTGGATCCTTCAGAGTCCGTTG
Swan goose *slc26a12p*	GCCATGGCTACTTCACACCACAACTTCGCTGGATCCTTCAGAGTCCGTTG
Black swan *slc26a12p*	GCCATGGCCACTTCACCCCACAACTTCATTGGATCCTTCGGAGTCCTTTG

Common snapping turtle *slc26a12*	CCTGATGATTGGGGAAACTGTGAACCGGCAGCTTAACCTAGCAGGGTATG
Green sea turtle *slc26a12p*	CCTGATGATTGGGGAAACCGTGAACCGGCGGCTTCACCTATCAGGGTATG
Bengalese finch *slc26a12*	CCTGATGGTGGGGCAGTCGGTGACGCGCCAGCTGCAGCTGGCGGGCTACG
Eurasian eagle owl *slc26a12p*	CCTGATGCTTGGGCAGTCTGTGAACCGGCACCTCCAGCTAGCAGGGTACA
Northern spotted owl *slc26a12p*	CCTCATGATTGGGCAGTGTGTGAACCGGCACCTCCAGCTAGCAGGGTACA
Burrowing owl *slc26a12p*	CCTGATGATTGGGCAGTCTGTGAACCAGCACCTCCAGCTAGCAGGGCACA
Dalmatian pelican *slc26a12*	CCTGATGATTGGGCAGTCTGTGAACCGGCACCTCCAGCTAGCAGGGTACA
Little egret *slc26a12p*	CCTGATGATAGGGCAGTCTGTGAACCGGCACCTCCAGCTAGCAGGGTACA
Emperor penguin *slc26a12p*	CCTGATGATTGGGCAGTCTGTGACCTGGCACCTCCAGCTAGCAGGGTACA
Rockhopper penguin *slc26a12p*	CCTGATGATTGGGCAGTCTGTGACCTGGCACCTCCAGCTAGCAGGGTACA
Yellow-eyed penguin *slc26a12p*	CCTGATGATTGGGCAGTCTGTGACCTGGCACCTCCAGCTAGCAGGGTACA
Adelie penguin *slc26a12p*	CCTGATGATTGGGCAGTCTGTGACCTGGCACCTCCAGCTAGCAGGGTACA
Magellanic penguin *slc26a12p*	CCTGATGATTGGGCAGTCTGTGACCTGGCACCTCCAGCTAGCAGGGTACA
Chicken *slc26a12*	CCTGATGATTGGGCAGTCTGTGAATCGGCACCTCCAGCTAGCGGGGTATG
Mallard *slc26a12p*	CCTGATAATCAGGCAGTCTGTGAACCGGCACCTCCAGCTAGCAGGGTACA
Duck *slc26a12p*	CCTGATAATCAGGCAGTCTGTGAACCGGCACCTCCAGCTAGCAGGGTACA
Muscovy duck *slc26a12p*	CCTGATGATCGGGCAGTCTGTGAACTGGCACCTCCAGCTAGCAGGGTACA
Pink-footed goose *slc26a12p*	CCTGATGATCGGGCAGTCTGTGAACCGGCAGCTCCAGCTAGCAGGGTACA
Swan goose *slc26a12p*	CCTGATGATCGGGCAGTCTGTGAACCGGCAGCTCCAGCTAGCAGGGTACA
Black swan *slc26a12p*	CCTGATGATCGGGCAGTCTGTGAACCGGCACCTCCAGCTAGCAGGGTACA

Common snapping turtle *slc26a12*	GAGATGGTAATAGCGGCTCTCCATCATTAATGAATTCCACATTGCCCAGC
Green sea turtle *slc26a12p*	GAGATGGTAATACCGGCTCTGCATCGTTAATGAATTCCACATTGCCCAGC
Bengalese finch *slc26a12*	GGGA---CAGCAG---CGCTGCCCTCGGGGACAACTCCAGCTCCCCCAGG
Eurasian eagle owl *slc26a12p*	GTGA---CAACGCTGGCTCTTCACGGGTGGGCAACTCCACCTCCTCCAGT
Northern spotted owl *slc26a12p*	GTGA---CAACGCTGGCTCTTCACTGGTGGGCAACTCCACCTCCTCCAGT
Burrowing owl *slc26a12p*	GTGA---CAACGCTGGCTCCTCACTGGTGGGCAACTCCACCTCCTCCAGT
Dalmatian pelican *slc26a12*	GTGAGGACAATGCTGGCTCTTCGCTGGTGGGCAACTCCAGCTTCTCCAGT
Little egret *slc26a12p*	GCGATGACAGCACTGGCTCTTTGCTGGTGGGCAACTCCACCTCTGCCGGT
Emperor penguin *slc26a12p*	GCGACGACAACTCCGGCTCTTTGCTGGTGGGCAACTCCACCTCCTCCAGT
Rockhopper penguin *slc26a12p*	GCGACAACA-CTCCGGCTCTTTGTTGGTGGGCAACTCCACCTCCTTCAGT
Yellow-eyed penguin *slc26a12p*	GCGACGACA-CTCCGGCTCTTTGTTGGTGGGCAACTCCACCTCCTTCAGT
Adelie penguin *slc26a12p*	GCGACGACAACTCCGGCTCTTTGCTGGTGGGCAACTCCACCTCCTCCAGT
Magellanic penguin *slc26a12p*	GCGATGACAACTCCG-CTCTTTGCTGGTGGGCAACTCCACCTCCTCCAGT
Chicken *slc26a12*	---ACGACAACACTGACTCTCTGCTGGTAGGCAATGCCACCTCCTCCGGT
Mallard *slc26a12p*	ACGACGGCAGCACTGGCTCTGTGCTGGTGGACAATTCCACCTTCCCTGGG
Duck *slc26a12p*	ACGACGGCAGCACTGGCTCTGTGCTGGTGGACAATTCCACCTTCCCTGGG
Muscovy duck *slc26a12p*	ACGACGGCAGCACTGGCTCTGTGCTGGTGGACAATTCCACCTCCCCTGGG
Pink-footed goose *slc26a12p*	GCGATGGCAGCACTGGCTCTGTGATGGTGGGCAATTCCACCTCCCCTGGG
Swan goose *slc26a12p*	GCGATGGCAGCACTGGCTCTGTGATGGTGGGCAATTCCACCTCCCCTGGG
Black swan *slc26a12p*	GCGACGGCAGCACTGGCTCTGTGCTGGTGGGCAATTCCACCTCCCCCGGG

Common snapping turtle *slc26a12*	AATTGG-ACAGCCATCTGTGACAGAAGCTGCTATGCAATCACTGTGGCAA
Green sea turtle *slc26a12p*	AACGGG-ACAGTCATCTGTGACAGAAGCTGCTATGCAATCACTGTGGCAA
Bengalese finch *slc26a12*	AACGGG-ACGGAGCTCTGTGACAGGAGCTGCTACGCCATCACCGTGGCCC
Eurasian eagle owl *slc26a12p*	AACGGG-ACAGGAGCCTGTGACAGGAGCTGCTACGCCATCTCTGTGGCCC
Northern spotted owl *slc26a12p*	AACGGG-ACAGGTGCCTGTGTCAGGAGCTGCTATGCCATCTCTGTGGCCC
Burrowing owl *slc26a12p*	AACGGG-ACAGGTACCTGTGACAGGAGCCGCTACACCATCTCCGTGGCCT
Dalmatian pelican *slc26a12*	AACGGG-ACGGGTGCCTGTGACAGGAGCTGCTACGCCATCACTGTGGCCC
Little egret *slc26a12p*	AATGGG-GTGGGTGCCTGTGACAGGAGCTGCTACGCCATCACTGTGGCCC
Emperor penguin *slc26a12p*	AACGGG-ACAGATGCCTGTTGCCGGTGCTGGTAGGTCATCACTGTGGCCC
Rockhopper penguin *slc26a12p*	AACGGG-ACAGGTGCCTGTGACAGGAGCTGCTACGCCATCACTGTGGCCC
Yellow-eyed penguin *slc26a12p*	AACGGG-ACAGGTGCCTGTGACAGGAGCTGCTACGCCATCACTGTGGCCC
Adelie penguin *slc26a12p*	AACAGG-ACAGGTGCCTGTGACAGGAGCTGCTACGCCATCACTGTGGCCC
Magellanic penguin *slc26a12p*	AACGGG-ACAGGTGCCTGTGACAGGAGCTGCTACGCCATCACTGTGGCCC
Chicken *slc26a12*	AACGGG-ACGGGTACCTGTGACAGGAGCTGCTACGCCATCACTGTGGCCC
Mallard *slc26a12p*	AACGGGGATGGCTACCTGTGACAGGAGCTGCTA--------CTGTGGCCC
Duck *slc26a12p*	AACGGGGATGGCTACCTGTGACAGGAGCTGCTA--------CTGTGGCCC
Muscovy duck *slc26a12p*	AATGGG-ATGGCTACCTGTGACAGGAGCTGCTA--------CTGTGGCCC
Pink-footed goose *slc26a12p*	AACGGG-ATGGCTACCTGTGACAAGAGCTGCTACGCCATCACTGTGGCCC
Swan goose *slc26a12p*	AACGGG-ATGGCTACCTGTGACAAGAGCTGCTACGCCATCACTGTGGCCC
Black swan *slc26a12p*	AACGGG-ACGGACACCTGTGACAAGAGCTGCTACGCCATCACTGTGGCCC

Common snapping turtle *slc26a12*	TTTCCTTAACTTTCCTTGTTGGGCTTTACCAG
Green sea turtle *slc26a12p*	TTTCCTTAACTTTCCTTGTTGGGCTTTACCAG
Bengalese finch *slc26a12*	TGTCCTTGAGCTTTCTGGTGGGCCTTTACCAG
Eurasian eagle owl *slc26a12p*	TTTCCTTAAGCTTTGTGGTCAGTCTTTACCAG
Northern spotted owl *slc26a12p*	TTTCCTTAAGCTTTCTGGTCAGTCTTTACCAG
Burrowing owl *slc26a12p*	TTTTCTTAAGCTTTCTGGTCAGTCTTTACCAG
Dalmatian pelican *slc26a12*	TTTCCTTAAGCTTTCTGGTCGGTCTTTACCAG
Little egret *slc26a12p*	TTTCCTTAAGCTTTCTGGTTGGTCTTTACCAG
Emperor penguin *slc26a12p*	TTTCCTTAAGCTTTCTGGTCGGTCTTTACCAG
Rockhopper penguin *slc26a12p*	TTTCCTTAAGCTTTCTGGTCGGTCTTTACCAG
Yellow-eyed penguin *slc26a12p*	TTTCCTTAAGCTTTCTGGTCGGTCTTTACCAG
Adelie penguin *slc26a12p*	TTTCCTTAAGCTTTCTGGTTGGTCTTTACCAG
Magellanic penguin *slc26a12p*	TTTCCTTAAGCTTTCTGGTCAGTCTTTACCAG
Chicken *slc26a12*	TTTCCTTGAGCTTTCTGGTTGGTCTTTACCAG
Mallard *slc26a12p*	TTTCCCTGAGCTTTCTGGTTGGCCTTTACCAG
Duck *slc26a12p*	TTTCCCTGAGCTTTCTGGTTGGCCTTTACCAG
Muscovy duck *slc26a12p*	TTTCCCTGAGCTTTCTGGTTGGCCTTTACCAG
Pink-footed goose *slc26a12p*	TTTCCCTGAGCTTTCTGGCTGGCCTTTACCAG
Swan goose *slc26a12p*	TTTCCCTGAGCTTTCTGGCTGGCCTTTACCAG
Black swan *slc26a12p*	TTTCTCTGAGCTTTCTGTTTGGCCTTTACCAG

B Exon2

Common snapping turtle *slc26a12*	ATCCTTCTAGGTGTTTTCCAGCTGGGCTTCATAGCTGTGTACCTGTCAGA
Green sea turtle *slc26a12p*	ATCCTGCTGGGGGTTTTACAGCTGGGCTTTGTGGCTGTATACCTGTCAGA
Dalmatian pelican *slc26a12*	ATCCTGCTGGGGGTTTTACAGCTGGGCTTTGTGGCTGTCTACCTGTCAGA
Little egret *slc26a12p*	ATCCTGCTGGGGGTTTTACAGCTGGGCTTTGTGGCTGTATACCTGTCAGA
Emperor penguin *slc26a12p*	ATCCTGCTGGGGGTTTTACAGCTGGGCTTTGTGGCTGTCTACCTGTCAGA
Rockhopper penguin *slc26a12p*	ATCCTGCTGGGGGTTTTACAGCTGGGCTTTGTGGCTGTCTACCTGTCAGA
Yellow-eyed penguin *slc26a12p*	ATCCTGCTGGGGGTTTTACAGCTGGGCTTTGTGGCTGTCTACCTGTCAGA
Adelie penguin *slc26a12p*	ATCCTGCTGGGGGTTTTACAGCTGGGCTTTGTGGCTGTCTACCTGTCAGA
Magellanic penguin *slc26a12p*	ATCCTGCTGGGGGTTTTACAGCTGGGCTTTGTGGCTGTCTACCTGTCAGA
Chicken *slc26a12*	ATCCTGCTGGGGGTTTTACAGCTGGGCTTTCTGTCTGTCTACCTGTCAGA
Mallard *slc26a12p*	ATCCTGCTGGGGGTTTTACAGCTGGGCTTTGTGGCTGTCTACCGGTCAGA
Duck *slc26a12p*	ATCCTGCTGGGGGTTTTACAGCTGGGCTTTGTGGCTGTCTACCGGTCAGA
Muscovy duck *slc26a12p*	ATCCTGCTGGGGGTTTTGCAGCTGGGCTTTGTGGCTGTCTACCGGTCAGA
Pink-footed goose *slc26a12p*	ATCCTGCTGGGGGGTTTACAGCTGGGCTTTGTGGCTGTCTACCTGTCAGA
Swan goose *slc26a12p*	ATCCTGCTGGGGGGTTTACAGCTGGGCTTTGTGGCTGTCTACCTGTCAGA
Black swan *slc26a12p*	ATCCTGCTGGGGGATTTACAGCTGGGCTTTGTGGCTGTCTACCTGTCAGA

Common snapping turtle *slc26a12*	ACCGCTCCTGAGTGGCTTCGTGACCGGAGCCTCTCTGACCATCCTCACCT
Green sea turtle *slc26a12p*	ACCTCTGCTCAGTGGCTTTGTGACTGGCTCCAGACTCACCATCATCACCT
Dalmatian pelican *slc26a12*	ACCTCTCCTCAGTGGCTTTGTGACCAGCTCCAGCCTCACCATTATCACCT
Little egret *slc26a12p*	ACCTCTGCTCAGTGGCTTTGTGACTGGCTCCAGACTCACCATCATCACCT
Emperor penguin *slc26a12p*	ACCTCACCTCAGTGGCTTTGTGACCGGATCTAACCTCACCATTATCACCT
Rockhopper penguin *slc26a12p*	ACCTCACCTCAGTGGCTTTGTGACCGGATCTAACCTCACCATTATCACCT
Yellow-eyed penguin *slc26a12p*	ACCTCACCTCAGTGGCTTTGTGACCGGATCTAACCTCACCATTATCACCT
Adelie penguin *slc26a12p*	ACCTCACCTCAGTGGCTTTGTGACTGGATCTAACCTCACCATTATCACCT
Magellanic penguin *slc26a12p*	ACCTCACCTCAGTGGCTTTGTGACCGGATCTAACCTCACCATTATCACCT
Chicken *slc26a12*	ACCTCTCCTCAGCGGCTTTGTGGCCGGCTCCAGCCTCACCATCATCACCT
Mallard *slc26a12p*	ACCTCTCCTCAGTGGCTTTGTGACCAGCTCCAGCCTCACCATTATCACCT
Duck *slc26a12p*	ACCTCTCCTCAGTGGCTTTGTGACC------AGCCTCACCATTATCACCT
Muscovy duck *slc26a12p*	ACCTCTTCTCAGTGGCTTTGTGACCAGCTCCAGCCTCACCGTTATCACCT
Pink-footed goose *slc26a12p*	ACCTCTCCTCAGTGGCTTTGTGACCGGCTCCAGCCTCACCATTATCATCT
Swan goose *slc26a12p*	ACCTCTCCTCAGTGGCTTTGTGACCGGCTCCAGCCTCACCATTATCATCT
Black swan *slc26a12p*	ACCTCTCCTCAGTGGCTTTGTGACCAGCTCCAGCCTCACCATTATCACCT

Dalmatian pelican *slc26a12*	CCCAAATGAAGTATCTCCTGGGACTGAAAATCCCTCGTCACGAAGGGGTG
Yellow-eyed penguin *slc26a12p*	CCCAGATGAAGTATCTCCTGGGACTAAAAATCCCTCGTCATGAAGGGGTG
Adelie penguin *slc26a12p*	CCCAGATGAAGTATCTCCTGGGACTAAAAATCCCTCGTCATGAAGGGGTG
Chicken *slc26a12*	CTCAGATGAAGTACCTCCTCGGGCTGAATATTCCCCGGCACGAAGGGGTG
Muscovy duck *slc26a12p*	CCCAGATGAAGTACCTCCTGGGACTGAAAATCCCTTGGCACGAAGGGGTT
Black swan *slc26a12p*	CCCAGATGAAGTACCTCCTGGGACTGAAAATCCCTCGGCAAGAAGGGGTG
Mallard *slc26a12p*	CCCAGATGAAGTACCTCCTGGGACTGAAAATCCCTTGTCATGAAGGTGTG
Pink-footed goose *slc26a12p*	CCCAGATGAAGTACCTCCTGGGACTGAAAATCCCTCGGCACGAAGGGGTG
Duck *slc26a12p*	CCCAGATGAAGTACCTCCTGGGACTGAAAATCCCTTGTCATGAAGGTGTG
Swan goose *slc26a12p*	CCCAGATGAAGTACCTCCTGGGACTGAAAATCCCTCGGCACGAAGGGGTG
Green sea turtle *slc26a12p*	CCCAAATAAAGTATCTCCTGGGACTGAAAATCCCTCGCCATGAAGGGGTG
Emperor penguin *slc26a12p*	CCCAGATAAAGTATCTCCTGGGACTAAAAATCCCTCGTCATGAAGCGGTG
Little egret *slc26a12p*	CCCAAATAAAGTATCTCCTGGGACTGAAAATCCCTCGCCATGAAGGGGTG
Magellanic penguin *slc26a12p*	CCCAGATGAAGTATCTCCTGGGACTAAAAATCCCTCATCATGAAGGGGTG
Rockhopper penguin *slc26a12p*	CCCAGATGAAGTATCTCCTGGGACTAAAAATCCCTCGTCATGAAGGGGTG
Common snapping turtle *slc26a12*	CTCAGATGAAGTATCTTCTTGGGTTGAAAATCCCTCGCAAGGAGGGGGTG

Common snapping turtle *slc26a12*	GGATCCCTCATCCTGACATGGGTTGAGATCTTCCGGTACATTCAGAAAAC
Green sea turtle *slc26a12p*	GGGTCCTTCATCCTGACGTGGGTTGATCTTTTCAGATACATCCAGAACAC
Dalmatian pelican *slc26a12*	GGGTCCTTTATCCTGACGTGGGTTGACCTTTTCAGATACATCCAGAACAC
Little egret *slc26a12p*	GGGTCCTTCATCCTGACGTGGGTTGATCTTTTCAGATACATCCAGAACAC
Emperor penguin *slc26a12p*	GGGTCCTTCATCCTGACATAGGTTGACCTTTTCAGATACATCCAGAACAC
Rockhopper penguin *slc26a12p*	GGGTCCTTCATCCTGACGTAGGTTGACCTTTTCAGATACATCCAGAACAC
Yellow-eyed penguin *slc26a12p*	GGGTCCTTCATCCTGACGTAGGTTGACCTTTTCAGATACATCCAGAACAC
Adelie penguin *slc26a12p*	GGGTCCTTCATCTTGACGTAGGTTGACCTTTTCAGATACATCCAGAACAC
Magellanic penguin *slc26a12p*	GGGTCCTTCATCCTGACGTAGGTTGACCTTTTCAGATACATCTAGAACAC
Chicken *slc26a12*	GGGTCCTTCATCCTGACTTGGGTGGACCTTTTCAGGTACATCCCGAACAC
Mallard *slc26a12p*	GGCTCCTTCATCCTGATGTGGCTTGACCTTTTCAGGTACATCCAGAACAC
Duck *slc26a12p*	GGCTCCTTCATCCTGATGTGGCTTGACCTTTTCAGGTACATCCAGAACAC
Muscovy duck *slc26a12p*	GG------CTTGACCTTTCAGGTTGACCTTTTCAGGTACATCCAGAACAC
Pink-footed goose *slc26a12p*	GGCTCCTTCATCCTGACGTGGCTTGACCTTTTCAGGTACATCCAGAACAC
Swan goose *slc26a12p*	GGCTCCTTCATCCTGACGTGGCTTGACCTTTTCAGGTACATCCAGAACAC
Black swan *slc26a12p*	GGCTCCTTCATCCTGACGTGGCTTGACCTTTTCAGGTACATCCAGAACAC

Common snapping turtle *slc26a12*	CAACATCTGTGACCTGGTCACCAGCCTGATTGCTTTGGCCATCATTGTGC
Green sea turtle *slc26a12p*	CAACATCTGTGACCTGGTCACCAGCCTGATTGCTTTTGCCATTATAATGC
Dalmatian pelican *slc26a12*	CAACATCTGTGACCTGGTCACCAGCCTAGTTGCTTTGGCCATCATAGTGC
Little egret *slc26a12p*	CAACATCTGTGACCTGGTCACCAGCCTGATTGCTTTTGCCATTATAATGC
Emperor penguin *slc26a12p*	CAGCATCTGTGACCTGGTCACTAGCCTGGTTGCTTTGGCCATCATAGTGC
Rockhopper penguin *slc26a12p*	CAGCATCTGTGACCTGGTCACTAGTCTGGTTGCTTTGGCCATCATAGTGC
Yellow-eyed penguin *slc26a12p*	CAGCATCTGTGACCTGGTCACTAGTCTGGTTGCTTTGGCCATCATAGTGC
Adelie penguin *slc26a12p*	CAGCATCTGTGACCTGGTCACTAGCCTGGTTGCTTTGGCCATCATAGTGC
Magellanic penguin *slc26a12p*	CAGCATCTGTGACCTGGTCACTAGTCTGGTTGCTTTGGCCATCATAGTGC
Chicken *slc26a12*	CAATATCTGTGACCTGGTCACCAGCCTGGTGGCTTTGGCCATCATAATAC
Mallard *slc26a12p*	CAACATCTGCGACCTGCTCACCAGCCTCATTGCTTTGGCCATCATAGTGC
Duck *slc26a12p*	CAACATCTGCGACCTGCTCACCAGCCTCATTGCTTTGGCCATCATAGTGC
Muscovy duck *slc26a12p*	CAACATCTGCGACCTGCTCACCAGCCTCATTGCTTTGGCCATCATAGTGC
Pink-footed goose *slc26a12p*	CAACATCTGCAACCTGCTCATCAGCCTCGTTGCTTTGGCCATCATAGTGC
Swan goose *slc26a12p*	CAACATCTGCAACCTGCTCATCAGCCTCGTTGCTTTGGCCATCATAGTGC
Black swan *slc26a12p*	CAACATCTGCGACCTGCTCACCAGCCTCGTTGCTTTGGCCATCATAGTGC

Common snapping turtle *slc26a12*	CAGTCAAGGAGATCAATGTCCGGTACAAGGACAAGCTGAAGGCCCCGTTT
Green sea turtle *slc26a12p*	CTGTCAAAGAGATCAATGACCGGTATAAGGAGAAGATGAAGGCCCCATTC
Dalmatian pelican *slc26a12*	CTGTCAAAGAAATCAATGACCGGTATAAGGAGAAGATGAAGGCCCCATTC
Little egret *slc26a12p*	CTGTCAAAGAGATCAATGACCGGTATAAGGAGAAGATGAAGGCCCCATTC
Emperor penguin *slc26a12p*	CTGTCAAAGAGATCGATGACCAGTATAAGGAGAAGATGAAGGCCCCATTC
Rockhopper penguin *slc26a12p*	CTGTCAAAGAGATCGATGACCAGTATGAGGAGAAGATGAAGGCCCCATTC
Yellow-eyed penguin *slc26a12p*	CTGTCAAAGAGATCGATGACCAGTATGAGGAGAAGATGAAGGCCCCATTC
Adelie penguin *slc26a12p*	CTGTCAAAGAGATCGATGACCAGTATAAGGAGAAGATGAAGGCCCCATTC
Magellanic penguin *slc26a12p*	CTGTCAAAGAGATCGATGACCAGTATGAGGAGAAGATGAAGGCCCCATTC
Chicken *slc26a12*	CTGTCAAAATGATCAACGATCGGTACAAGGACAAGATGAAGGCCCCGGTT
Mallard *slc26a12p*	CTGTCAAAGAGCTCAATGACCGGTGTAAGGACAGGATGAAAGCCCTGTTC
Duck *slc26a12p*	CTGTCAAAGAGCTCAATGACCGGTGTAAGGACAGGATGAAAGCCCTGTTC
Muscovy duck *slc26a12p*	CTGTCAAAGAGCTCAATGACCAGTGTAAGGACGGGATGAAAGCCCCGTTC
Pink-footed goose *slc26a12p*	CTGTCAAAGAGATCAATGACCGGTATAAGGACAGGATGAAGGCCCCGTTC
Swan goose *slc26a12p*	CTGTCAAAGAGATCAATGACCGGTATAAGGACAGGATGAAGGCCCCGTTC
Black swan *slc26a12p*	CTGTCAAAGAGATCAATGACCAGTATAAGGACAGGGTGAAGGCCCCGTTC

Common snapping turtle *slc26a12*	CCTGTGGAGCTGCTAGTGGTCATTGCTGGCACTT-----TGTTCTCCTAT
Green sea turtle *slc26a12p*	CCTGTAGAGCTGCTGGTGGTCATTGTAGCCACAG-----TAATATCTTAC
Dalmatian pelican *slc26a12*	CTCATAGAGCTGCTGGTGGTCATTGTAGCCACAG-----TAATATCTTAC
Little egret *slc26a12p*	CCTGTAGAGCTGCTGGTGGTCATTGTAGCCACAG-----TAATATCTTAC
Emperor penguin *slc26a12p*	CCCATAGAGCTGCTGGTGGTCATTGTAGCCACAG-----TAATATCTTAT
Rockhopper penguin *slc26a12p*	CCCATAGAGCTGCTGGTGGTCATTGTAGCCACAG-----TAGTATCTTAT
Yellow-eyed penguin *slc26a12p*	CCCATAGAGCTGCTGGTGGTCATTGTAGCCACAG-----TAATATCTTAT
Adelie penguin *slc26a12p*	TCCATAGAGCTGCTGGTGGTCATTGTAGCCACAG-----TAATATCTTAT
Magellanic penguin *slc26a12p*	CCCATAGAGCTGCTGGTGGTCATTGTAGCCACAG-----TAATATCTTAT
Chicken *slc26a12*	CCCATAGAGCTGCTCGTGGTCATCATAGCCACAG-----TAGTATCTTAC
Mallard *slc26a12p*	CCCATACAGCTGCTTGTGGTCATTGTAGCCACGG-----TGGTATCTTAC
Duck *slc26a12p*	CCCATACAGCTGCTTGTGGTCATTGTAGCCACGG-----TGGTATCTTAC
Muscovy duck *slc26a12p*	CCCATAGAGCTGCTTGTGGTCATTGTAGCCACGG-----TGGTATCTTAC
Pink-footed goose *slc26a12p*	CCCATAGAGCTGCTTGTGGTCATTGTAGCCACGG-----TGGTATCTTAC
Swan goose *slc26a12p*	CCCATAGAGCTGCTTGTGGTCATTGTAGCCACGG-----TGGTATCTTAC
Black swan *slc26a12p*	CCCATAGAGCTGCTTTTGGTCATTGCAGTATCTTACTACTGGTATCTTAC

Common snapping turtle *slc26a12*	TATTTTAAGTTTGAGAAGCAATACAATTCTGCCATTTGTGGGAGCATCCC
Green sea turtle *slc26a12p*	TACTTTAACTTCGAAGAGTAATACAAGTCTGCTGTTTGTGGGGATATCCC
Dalmatian pelican *slc26a12*	TACTTTAACTTCGAAGAGCAATACAAGTCTGCTGTTTGTGGGGATATCCC
Little egret *slc26a12p*	TACTTTAACTTCGAAGAGTAATACAAGTCTGCTGTTTGTGGGGATATCCC
Emperor penguin *slc26a12p*	TACTTTAACTTCGAAGAGCGATACAAGTCTGCTGTTAGGGG--ATATCCC
Rockhopper penguin *slc26a12p*	TACTTTAACTTCGAAGAGCAATACAAGTCTTCTGTTAGGGG--ATATCCC
Yellow-eyed penguin *slc26a12p*	TACTTTAACTTCGAAGAGCGATACAAGTCTGCTGTTAGGGG--ATATCTC
Adelie penguin *slc26a12p*	TACTTTAATTTTGAAGAGCGATACAAGTCTGCTGTTAGGGG--ATATCCC
Magellanic penguin *slc26a12p*	TACTTTAACTTTGAAGAGCGATACAAGTCTGCTATTAGGGG--ATATCCC
Chicken *slc26a12*	TACTTTAAATTCGAAGAGCGATACAAATCTTCAGTTTGTGGGGCCATCCC
Mallard *slc26a12p*	TACTTCAACTTTCAAGA-----ACAAGTTGGCTGTTTGTGGGGCTATCCC
Duck *slc26a12p*	TACTTCAACTTTCAAGA-----ACAAGTTGGCTGTTTGTGGGGCTATCCC
Muscovy duck *slc26a12p*	TACTTCAACTTTCAAGAGCGATACAAGTTGGCTGTTTGTGGGGCTATCCC
Pink-footed goose *slc26a12p*	TACTTCAACTTTCAAGAGTGATACAAGTCGGCTGTTTGTAGGGCTATCCC
Swan goose *slc26a12p*	TACTTCAACTTTCAAGAGTGATACAAGTCGGCTGTTTGTAGGGCTATCCC
Black swan *slc26a12p*	TACTTCAACTTTCAAGAGCGATACAAGTCAGCCGTTTGTGGGGCTATCCC

Common snapping turtle *slc26a12*	CACTGGTTTCCAGAAACCCACCATACCAGACCCGCACCTGTTTTCCAGTC
Green sea turtle *slc26a12p*	CACTGGTTTCAGGAAACCCACTCTACCAGACATAAACCTGTTTTCCAGCC
Dalmatian pelican *slc26a12*	CACCGGTTTCAGGAAACCCACTCTACCAGATATAAACCTGTTTTCCAGCC
Little egret *slc26a12p*	CACTGGTTTCAGGAAACCCACTCTACCAGACATAAACCTGTTTTCCAGCC
Emperor penguin *slc26a12p*	CACTGGTTTCAGGAAACCCACTCTACCAGATACAAACCTGTTTTCCAGCC
Rockhopper penguin *slc26a12p*	CACTGGTTTCAGGAAACCCACTCTACCAGATATAAACCTGTTTTCCAGCC
Yellow-eyed penguin *slc26a12p*	CACTGGTTTCAAGAAACCCACTCTACCAGATATAAACCTGTTTTCCAGCC
Adelie penguin *slc26a12p*	CACTGGTTTCAGGAAACCCACTCTACCAGATATAAACCTGTTTTCCAGCC
Magellanic penguin *slc26a12p*	CACTGGTTTCAGGAAACCCACTCTACCAGATATAAACCTGTTTTCCAGCC
Chicken *slc26a12*	CACTGGTTTCAGGAAACCCACCCTACCAGACACAAAGCTGTTTTCCAGCC
Mallard *slc26a12p*	CACTGGTTTCAGGAAGCCCACTCTGCCAGATACAAAGCCGTTCTCCAGCT
Duck *slc26a12p*	CACTGGTTTCAGGAAGCCCACTCTGCCAGATACAAAGCCGTTCTCCAGCT
Muscovy duck *slc26a12p*	CACTGGTTTCAGGAAGCCCACTCTGCCAGATACGAAGCCGTTCTCCAGCT
Pink-footed goose *slc26a12p*	CACTGGTTTCAGGAAGCCCACCCTACCAGATATGAAGCTGTTCTCCAGCC
Swan goose *slc26a12p*	CACTGGTTTCAGGAAGCCCACCCTACCAGATATGAAGCTGTTCTCCAGCC
Black swan *slc26a12p*	CACTGGTTTCAGGAAGCCCACCCTACCAGATATGAAGCTGTTCTCCAGCC

Common snapping turtle *slc26a12*	TCGCAGTTGATGCTGTGCCCATTGCTATCATTGGCTTCGCCACGACAGTC
Green sea turtle *slc26a12p*	TGGCAGTTGATGCTCTGCCCATTGCCGTTATTGGCTTTGCCATGACTGTT
Dalmatian pelican *slc26a12*	TGGCAGTTGATGCTCTGCCGATTGCTGTTATTGGCTTTGCCATGACTGTG
Little egret *slc26a12p*	TGGCAGTTGATGCTCTGCCCATTGCCGTTATTGGCTTTGCCATGACTGTT
Emperor penguin *slc26a12p*	TGGCAGTTGATGCTCTGCCCATTGCTGTTATTGGCTTTGCCATGATTGTC
Rockhopper penguin *slc26a12p*	TGGCAGTTGATTCTCTGCCCATTGCTGTTACTAGCTTTGCCGTGATTGTC
Yellow-eyed penguin *slc26a12p*	TGGCAGTTGATGCTCTGCCCATTGCTGTTACTAGCTTTGCCGTGATTGTC
Adelie penguin *slc26a12p*	TGGCAGTTGATGCTCTGCCCATTGCTGTTACTGGCTTTGCCGTGATTGTC
Magellanic penguin *slc26a12p*	TGGCAGTTGATGCTCTGCCCATTGCTGTTACTAGCTTTGCCGTGATTGTC
Chicken *slc26a12*	TGGCAGTCGATGCTCTGCCCATTGCTATTATAGGCTTTGCTATGACTGTC
Mallard *slc26a12p*	TGGCAGTCGATGCCCTGCCCATTGCTATTATTGGCTTTGCTATGACCGTC
Duck *slc26a12p*	TGGCAGTCGATGCCCTGCCCATTGCTATTATTGGCTTTGCTATGACCGTC
Muscovy duck *slc26a12p*	TGGCAGTCGATGCCCTGCCCATTGCTATTATTGGCTTTGCTATGACCATC
Pink-footed goose *slc26a12p*	TGGCAGTCGATGCCCAGCCCATTGCTATTATTGGCTTTGCTATGACCATC
Swan goose *slc26a12p*	TGGCAGTCGATGCCCAGCCCATTGCTATTATTGGCTTTGCTATGACCATC
Black swan *slc26a12p*	TGACAGTCGATGCCCAGCCCATTGCTATTACTGGCTTTGCTATGACTGTC

Common snapping turtle *slc26a12*	TCCCTCGCAGAAATCTTTGCCAAAAAGCACGGCTACACTGTCCGAGCTAA
Green sea turtle *slc26a12p*	TCCCTGGTAGAAATCTTTGGCAAAAAGCATGGCTACACTGTCTGTGCCAA
Dalmatian pelican *slc26a12*	TCCCTGGCAGAAATCTTTGGCAAAAAGCATGGCTACGCTGTCCGTGCCAA
Little egret *slc26a12p*	TCCCTGGTAGAAATCTTTGGCAAAAAGCATGGCTACACTGTCTGTGCCAA
Emperor penguin *slc26a12p*	TCCCTGGTGGAAATCTTTGGCAAAAAGCACGGCTATGCTGTCCATGCCAA
Rockhopper penguin *slc26a12p*	TCCCTGGCAGAAATCTTTGGCAAAAAGCATGGCTACGCTGTCCATGCCAA
Yellow-eyed penguin *slc26a12p*	TCCCTGGCAGAAATCTTTGGCAAAAAGCATGGCTACGCTGTCCATGCCAA
Adelie penguin *slc26a12p*	TCCGTGGCGGAAATCTTTGGCAAAAAGCATGGCTACGCTGTCCATGCCAA
Magellanic penguin *slc26a12p*	TCCCTGGCAGAAATCTTTGGCAAAAAGCATGGCTACGCTGTCCATGCCAA
Chicken *slc26a12*	TCCCTGGCAGAAATCTTTGGCAAAAAGCATGGCTACGCCGTCCGTGCCAA
Mallard *slc26a12p*	TCCCTGGCAGAAATCTTTGGCAAAAAGCATGGCTACACCGTCTGTGCCAA
Duck *slc26a12p*	TCCCTGGCAGAAATCTTTGGCAAAAAGCATGGCTACACCGTCTGTGCCAA
Muscovy duck *slc26a12p*	TCCCTGGCAGAAATCTTTGGCAAAAAGCATGGCTACACCATCTGTGCCAA
Pink-footed goose *slc26a12p*	TCCCTGGCGGAAATCTTTGGCAAAAAGCACGGCTAAGCCGTCCGTGCCAA
Swan goose *slc26a12p*	TCCCTGGCGGAAATCTTTGGCAAAAAGCACGGCTAAGCCGTCCGTGCCAA
Black swan *slc26a12p*	TCCCTGGCGGAAATCTTTGGCAAAAAGCACCGCTACGCCGTCTGCGCCAA

Common snapping turtle *slc26a12*	CCAAGAGATGATCGCCATTGGCATGTGCAACCTGGTCCCCTCTTTCTTTC
Green sea turtle *slc26a12p*	CCACGAGATGATTGCCACTGGCATGCGCAACCTCATCCCTTCTTTCTTCT
Dalmatian pelican *slc26a12*	CCAAGAGATGATTGCCATTGGCATGTGCAACCTGATCCCTTCTTTCTTCT
Little egret *slc26a12p*	CCACGAGATGATTGCCACTGGCATGCGCAACCTCATCCCTTCTTTCTTCT
Emperor penguin *slc26a12p*	CCAAGAGATGATTGCCATTGGCATGTGCAACCTGATCCCTTCTTTCTTCT
Rockhopper penguin *slc26a12p*	CCAAGAGATGAGTGCCATTGGCATGTGCAACCTGATCCCTTCTTTCTTCT
Yellow-eyed penguin *slc26a12p*	CCAAGAGATGAGTGCCATTGGCATGTGCAACCTGATCCCTTCTTTCTTCT
Adelie penguin *slc26a12p*	CCAAGAGATGATTGCCATTGGCACGTGCAACCTGATCCCTTCTTTCTTCT
Magellanic penguin *slc26a12p*	CCAAGAGATGATTGCCATTGGCATGTGCAACCTGATCCCTTCTTTCTTCT
Chicken *slc26a12*	CCAAGAGATGATCGCCATTGGCATGTGCAACCTGGTCCCATCTTTCTTCT
Mallard *slc26a12p*	CCAAGAGATGATCGCCATTGGCATGTGCAACCTGATCCCAGCTTTCTTCT
Duck *slc26a12p*	CCAAGAGATGATCGCCATTGGCATGTGCAACCTGATCCCAGCTTTCTTCT
Muscovy duck *slc26a12p*	CCAAGAGATGATCGCCATTGGCATGTGCAACCTGATCCCAGCTTTCTTCT
Pink-footed goose *slc26a12p*	CCAAGAGCTGGTCGCCATTGGCATGTGCAACCTGATCCCAGCTTTCTTCT
Swan goose *slc26a12p*	CCAAGAGCTGGTCGCCATTGGCATGTGCAACCTGATCCCAGCTTTCTTCT
Black swan *slc26a12p*	CCAAGAGATGATCGCCATTGGCATGTGCAACCTGATCCCGGCTTTCTTCT

Common snapping turtle *slc26a12*	ACTGTTTTGCCAGCTCTGCAGCCTTGGCCAAGACCCTGCTTAAGGAATCC
Green sea turtle *slc26a12p*	ACTGCTTTGCCAGCTCTGCAGCCCTGAGCAAGACTCTGCTGAAGGAGTCC
Dalmatian pelican *slc26a12*	ACTGCTTTGCGAGCTCTGCGGCCCTGACCAAGACTCTGCTGAAGGAGTCC
Little egret *slc26a12p*	ACTGCTTTGCCAGCTCTGCAGCCCTGAGCAAGACTCTGCTGAAGGAGTCC
Emperor penguin *slc26a12p*	ACTGCTTTGCCAGCTCTGTGGCCCTGACCAAGACTG--CTGAAGGAGTCC
Rockhopper penguin *slc26a12p*	ACTGCTTTGCCAGCTCTGTGGCCCTGACCAAGAATG--CTGAAGGAGTCC
Yellow-eyed penguin *slc26a12p*	ACTGCTTTGCCAGCTCTGTGGCCCTGACCAAGAATG--CTGAAGGAGTCC
Adelie penguin *slc26a12p*	ACTGCTTTGCCAGCTCTGTGGTCCTGACCAAGACTCTGCTGAAGGAGTCC
Magellanic penguin *slc26a12p*	ACTGCTTTGCCAGCTCTGTGGCCCTGACCAAGAATG--CTGAAGGAGTCC
Chicken *slc26a12*	ACTGCTTTGCTAGCTCTGCAGCCTTGACCAAGACTCTCCTGAAGGAGTCC
Mallard *slc26a12p*	ACTGCTTTGCCAGCTCCGTGGCCTTGACCAAGACTCTGCTGAAGGAGTCC
Duck *slc26a12p*	ACTGCTTTGCCAGCTCCGTGGCCTTGACCAAGACTCTGCTGAAGGAGTCC
Muscovy duck *slc26a12p*	ACTGCTTTGCCAGCTCTGTGGCCTTGACCAAGACTCTGCTGAAGGAGTCC
Pink-footed goose *slc26a12p*	ACAGCTTTGCCAGCTCTGCAGCCTTGACCAAGACTCTGCTGAAGGAGTCC
Swan goose *slc26a12p*	ACAGCTTTGCCAGCTCTGCAGCCTTGACCAAGACTCTGCTGAAGGAGTCC
Black swan *slc26a12p*	ACTGCTTTGCCAGCTCTATGGCCTTGACCAAGACTCTGCTGAAGGAGTCC

Common snapping turtle *slc26a12*	ACAGGCTGCCACACCCAAATCTCAGGGCTGATAACCTCCGGGGTGCTGCT
Green sea turtle *slc26a12p*	ACTGGGACCCAGAGTCAGGTCTCTGGCCTGGTCACCTCCCTGGTGCTGCT
Dalmatian pelican *slc26a12*	ACGGGGACACAGACCCAGGTCTCTGGCCTGGTCACCTCCCTGGTGCTGCT
Little egret *slc26a12p*	ACTGGGACCCAGAGTCAGGTCTCTGGCCTGGTCACCTCCCTGGTGCTGCT
Emperor penguin *slc26a12p*	ATGGGGACCCAGACCCAGGTCTCTGGCCTAGTCACCTCC-TGGTGCTGCT
Rockhopper penguin *slc26a12p*	ACGGGGACCCAGACCCAGGTCTCTGGCCTAGTCACCTCC-TGGTGCTGCT
Yellow-eyed penguin *slc26a12p*	ACGAGGACCCAGACCCAGGTCTCTGGCCTAGTCACCTCC-TGGTGCTGCT
Adelie penguin *slc26a12p*	ACGGGGACCCAGACCCAGGTCTCTGGCCTAGTCACCTCC-TGGTGCTGCT
Magellanic penguin *slc26a12p*	ACGGGGACCCAGACCCAGGTCTCTGGCCTAGTCACCTCC-TGGTGCTGCT
Chicken *slc26a12*	ACAGGAACCCAAACGCAGCTCTCTAGCCTGGTCACCTCCCTGGTGCTGCT
Mallard *slc26a12p*	ACAGGGACCCAAACCCAGATCTTTGGCCTGGTCACCTCACTGGTGCTGCT
Duck *slc26a12p*	ACAGGGACCCAAACCCAGATCTTTGGCCTGGTCACCTCACTGGTGCTGCT
Muscovy duck *slc26a12p*	GCGGGGACCCAAACCCAGATCTCTGGCCTGGTCATCTCGCTGGTGCTGCT
Pink-footed goose *slc26a12p*	ACGGGGACCCAAATCCAGGTCTCTGGCCTGGTCACCTCCCTGGTGCTGCT
Swan goose *slc26a12p*	ACGGGGACCCAAATCCAGGTCTCTGGCCTGGTCACCTCCCTGGTGCTGCT
Black swan *slc26a12p*	ACGGGGACCCAAACCCAGGTCTCTGGCCTGGTCACCTCCCTGGTGCTGCT

Common snapping turtle *slc26a12*	ACTGGTGCTGCTGTGGATTTCCCCTCTCTTCTACTCGCTGCAGATCAGCA
Green sea turtle *slc26a12p*	GCTAGTGCTGCTGTGGATTGCCCTGCTCTTCTACTCGCTGCAGACCTCCA
Dalmatian pelican *slc26a12*	GCTAGTGCTGCTGTGGATTGCCCCGCTCTTCTACTCGTTGCAGACCTCCA
Little egret *slc26a12p*	GCTAGTGCTGCTGTGGATTGCCCTGCTCTTCTACTCGCTGCAGACCTCCA
Emperor penguin *slc26a12p*	GCTAGCGCTGCTGTGGATTGCCCTGCTCTTCTACTCGTTGCAGACCTCTA
Rockhopper penguin *slc26a12p*	GCTAGTGCTGCTGTGGATTGCCCTGCTCTTCTACTTGTTGCAGACCTCTA
Yellow-eyed penguin *slc26a12p*	GCTAGTGCTGCTGTGGATTGCCCTGCTCTTCTACTTGTTGCAGACCTCTA
Adelie penguin *slc26a12p*	GCTAGTGCTGCTGTGGATTGCCCTGCTCTTCTACTCGTTGCAGACCTCTG
Magellanic penguin *slc26a12p*	GCTACTGCTGCTGTGGATTGCCCTGCTCTTCTACTTGTTGCAGACCTCTA
Chicken *slc26a12*	GCTGGTGTTGCTGTGGATTGCTCCGCTCTTCTACTCTCTGCAAACCGCCA
Mallard *slc26a12p*	AATAGTGTTGCTGTGAATCGTGCCACTCTTCTACTCGCTGCAGACCTCCA
Duck *slc26a12p*	AATAGTGTTGCTGTGAATCGTGCCACTCTTCTACTCGCTGCAGACCTCCA
Muscovy duck *slc26a12p*	ACTAGTGTTGCTGTGAATCGTGCCACTCTTCTACTCGCTGCAGACCTCCA
Pink-footed goose *slc26a12p*	GCTAGTGTTGCTGTGGATCGCGCCACTCTTCTACTCGCTGCAGACCTCCA
Swan goose *slc26a12p*	GCTAGTGTTGCTGTGGATCGCGCCACTCTTCTACTCGCTGCAGACCTCCA
Black swan *slc26a12p*	GCTAGTGTTGCTGTGGATCGTGCCGCTCTTCTACTTGCTGCAGACCTCCA

Common snapping turtle *slc26a12*	TCCTGGGGGTGATCACCATCGCCAACCTTCGGGGAGGCTTGCGGAAGTTT
Green sea turtle *slc26a12p*	TCCTGGGGGTGGTCACTATTGTCAACCTGCAGGGGGGGCCTGAGGAGGTTC
Dalmatian pelican *slc26a12*	TCCTGGGGGTGGTCACTATTGTCAACCTGCGGGGGGGCCTGAGGAAGTTC
Little egret *slc26a12p*	TCCTGGGGGTGGTCACTATTGTCAACCTGCAGGGGGGGCCTGAGGAGGTTC
Emperor penguin *slc26a12p*	TCCTGGGGGTGGTCACCGTTGTCAACCTGCAGAG----------------
Rockhopper penguin *slc26a12p*	TCCTGGGGGTGGTCACCATCGTCAGCCTGCGGGGTTGTGGTGGCTTGTGT
Yellow-eyed penguin *slc26a12p*	TCCTGGGGGTGGTCACCATTGTCAACCTGCGGGGTTGTGGTGGCTTGTGT
Adelie penguin *slc26a12p*	TCCTGGGGGTGGTCACGATTGTCAACCTGCAGGGTTGTGGTGGCTTGTGT
Magellanic penguin *slc26a12p*	TCCTGGGGGTGGTCACCATTGTCAACCTGCGGGGTTGTGGTGGCTTGTGT
Chicken *slc26a12*	TCCTGGGGGTGGTCACCATTGTCAACCTGCGGGGGGGCCTGAGGACGTTT
Mallard *slc26a12p*	TCCTGGGGGTGGGCACCATTGCCGACCTGCGGGGGG-CCTGAGGAGGTTC
Duck *slc26a12p*	TCCTGGGGGTGGGCACCATTGCCGACCTGCGGGGGG-CCTGAGGAGGTTC
Muscovy duck *slc26a12p*	TCCTGGGGGTGGGCACCATTGCCAACCTGCGGGGGG-CCTGAGGAGGTTC
Pink-footed goose *slc26a12p*	TCCTGGGGGTGGTCACCATTGCCAACCTGCGGGGGGGCCTGAGGAGGTTC
Swan goose *slc26a12p*	TCCTGGGGGTGGTCACCATTGCCAACCTGCGGGGGGGCCTGAGGAGGTTC
Black swan *slc26a12p*	TCCTGGGGGTGGTCACCATTGCCAACCTGCGGGGGGGCCTGAGGAGGTTC

Common snapping turtle *slc26a12*	GCTGACACACCCAGCATGTGGCGACTCAGCAAGGTGGATACGGTGGTCTG
Green sea turtle *slc26a12p*	CGTGACACCCCCGGCATGTGGCAGCTCAGCAAGCTGGACACGGTGGTGTG
Dalmatian pelican *slc26a12*	TGTGACACCCCCTGCATGTGGCAGCTCAGCAAGCTGGACATGGTGGTGTG
Little egret *slc26a12p*	CGTGACACCCCCGGCATGTGGCAGCTCAGCAAGCTGGACACGGTGGTGTG
Emperor penguin *slc26a12p*	--
Rockhopper penguin *slc26a12p*	TGTG--
Yellow-eyed penguin *slc26a12p*	TGT---
Adelie penguin *slc26a12p*	TGTG--
Magellanic penguin *slc26a12p*	TGT---
Chicken *slc26a12*	TGTGAAACCCCACGCATGTGGCAGCTCAGCAAGCTGGACACGGCGGTGTG
Mallard *slc26a12p*	TGCGACATCCCTGACATGTGGCAGCTCAGCAGGCTGGACACAGTTGTGTG
Duck *slc26a12p*	TGCGACATCCCTGACATGTGGCAGCTCAGCAGGCTGGACACAGTTGTGTG
Muscovy duck *slc26a12p*	TGCGACATCCCTGACATGTGGCAGCTCAGCAAGCTGGACACAGTGGTGTG
Pink-footed goose *slc26a12p*	TGCGACATCGCTTGCATATGGCAGCTCAGCAAGCTGGACACGGTGGTGTG
Swan goose *slc26a12p*	TGCGACATCGCTTGCATATGGCAGCTCAGCAAGCTGGACACGGTGGTGTG
Black swan *slc26a12p*	CGCGACATCCCTCGCATATGGCAGCTCAGCAAGCTGGACACGGTGGTGTG

Common snapping turtle *slc26a12*	GTGGGTCACCAT--GCTGGCCTCTTCGCTGATCTCGACAGAGATTGGACT
Green sea turtle *slc26a12p*	GTGGGCAACCATATGCTGTCCTCCACGCTGATCACCACGGAGATTGGGCT
Dalmatian pelican *slc26a12*	GTGGACAACCAT--GCTGTCCTCCACACTGATCACCACAGAGATTGGGCT
Little egret *slc26a12p*	GTGGGCAACCATATGCTGTCCTCCACGCTGATCACCACGGAGATTGGGCT
Emperor penguin *slc26a12p*	--
Rockhopper penguin *slc26a12p*	--
Yellow-eyed penguin *slc26a12p*	--
Adelie penguin *slc26a12p*	--
Magellanic penguin *slc26a12p*	--
Chicken *slc26a12*	GTGGACAACCAT--GCTGGCCTCCACGCTGATCACCACAGAGATAGGGCT
Mallard *slc26a12p*	GTAGACAACCAT--GCTGGCCTCCACGCTGATCACCACGGAGATCAGGCT
Duck *slc26a12p*	GTAGACAACCAT--GCTGGCCTCCACGCTGATCACCACGGAGATCAGGCT
Muscovy duck *slc26a12p*	GTGGACAACTAT--GCTGGCCTCCACGCTGATCACCATGGAGATCAGGCT
Pink-footed goose *slc26a12p*	GTGAACACCCAC--GCTGGCCTCCACACTGATCACCACGGAGATCGGGCT
Swan goose *slc26a12p*	GTGAACACCCAC--GCTGGCCTCCACACTGATCACCACGGAGATCGGGCT
Black swan *slc26a12p*	GTGGACAACCAT--GCTGGCCTCCACACTGGTCACCACGGAGATCGGGCT

Common snapping turtle *slc26a12*	CTTGGTAGGAGTCTGCTTCGCTCTCCTCTGCATCATCTTCCGCACCCAGA
Green sea turtle *slc26a12p*	CCTCGTGGGTGTCTGCTTCGCTATGCCCTGTATCATCTTCCGCACGCAGA
Dalmatian pelican *slc26a12*	CCTCGTGGGCGTCTGCGTCGCTCTGCTCTGTGTCATCTTCCGCACGCAGA
Little egret *slc26a12p*	CCTCGTGGGTGTCTGCTTCGCTATGCCCTGTATCATCTTCCGCACGCAGA
Emperor penguin *slc26a12p*	--
Rockhopper penguin *slc26a12p*	--
Yellow-eyed penguin *slc26a12p*	--
Adelie penguin *slc26a12p*	--
Magellanic penguin *slc26a12p*	--
Chicken *slc26a12*	CCTGGTGGGCGTCTGCTTTGCTCTGCTCTGCATCATCTTCCGCACGCAGA
Mallard *slc26a12p*	CCTTGTGGGCATCTGCTTTGCTCTGCTCTGCATCATCTTCCGCATGCAGA
Duck *slc26a12p*	CCTTGTGGGCATCTGCTTTGCTCTGCTCTGCATCATCTTCCGCATGCAGA
Muscovy duck *slc26a12p*	CCTCGTGGGCCTCTGCTTTGCTCTGCTCTGCATCATCTTCCGCATGCAGA
Pink-footed goose *slc26a12p*	CCATGTGGGCGTCTGCTTTGCTCTGCTCTGCATCATCTTCCACACACAGA
Swan goose *slc26a12p*	CCTTGTGGGCGTCTGCTTTGCTCTGCTCTGCATCATCTTCCACACACAGA
Black swan *slc26a12p*	CCTCGTGGGTGTCTGCTTTGCTCTGCTCTGCATCATCTTCCGCACGCAGA

Common snapping turtle *slc26a12*	GGCCCAGGGCCACCCTTCTGGGCAAGGTCAATGATACCGAAATTTATGAG
Green sea turtle *slc26a12p*	GGCCCAGGTCCACGCTCCTGGGCAAGGTCAGCAACATGGAAATCTATGAG
Dalmatian pelican *slc26a12*	GACCCAGGGCCATGCTCCTGGGCAAGGTCAGCAACACGCAAATCTATGAG
Little egret *slc26a12p*	GGCCCAGGTCCACGCTCCTGGGCAAGGTCAGCAACATGGAAATCTATGAG
Emperor penguin *slc26a12p*	--
Magellanic penguin *slc26a12p*	--
Rockhopper penguin *slc26a12p*	--
Yellow-eyed penguin *slc26a12p*	--
Adelie penguin *slc26a12p*	--
Chicken *slc26a12*	GACCCAGGGCTGCACTCCTGGGCAAGGTCAGCAACACGGAAATCTATGAA
Mallard *slc26a12p*	GACCCAGGGCCACGCTCCTGGGCAAGGTCAGCAACAC--AGATCTATGAG
Duck *slc26a12p*	GACCCAGGGCCACGCTCCTGGGCAAGGTCAGCAACAC--AGATCTATGAG
Muscovy duck *slc26a12p*	GATGCAGGGCCATGCTCCTGGGCAAGGTCAGCAACAC--AGATCTATGAG
Pink-footed goose *slc26a12p*	GACCCAGGGCCACGCTCCTGGGCAAGGTCAGCAACACGGAGATCTGTGAG
Swan goose *slc26a12p*	GACCCAGGGCCACGCTCCTGGGCAAGGTCAGCAACACGGAGATCTGTGAG
Black swan *slc26a12p*	GACCCAGGGCCACGCTCCTGGGCAAGGTTGGCAACACGGAGATCTACAAG

Common snapping turtle *slc26a12*	GACCAGTTCACTTACAAGCAGCTCAGCAGTATTACCAATGTCAAGATCTT
Green sea turtle *slc26a12p*	GACCAGTCCACTTACAAGAAGCTCAGCAGTATTGCCAACATCAAAATCTT
Dalmatian pelican *slc26a12*	GACCAGTCCACTTACAAGCAGCTCAGCAGTATTGCCAACATCAAAATCTT
Little egret *slc26a12p*	GACCAGTCCACTTACAAGAAGCTCAGCAGTATTGCCAACATCAAAATCTT
Emperor penguin *slc26a12p*	--
Rockhopper penguin *slc26a12p*	--
Yellow-eyed penguin *slc26a12p*	--
Adelie penguin *slc26a12p*	--
Magellanic penguin *slc26a12p*	--
Chicken *slc26a12*	GACCAGTCTGCTTACAAGCAGCTCAGCAGCATTGCCAACATCAAAATCTT
Mallard *slc26a12p*	GACCAGACCACCTACAGGCAACTCAGCAGTATTGCCAACATCAAAATCTT
Duck *slc26a12p*	GACCAGACCACCTACAGGCAACTCAGCAGTATTGCCAACATCAAAATCTT
Muscovy duck *slc26a12p*	GACCAGGCCACCTACAAGCAACTCAGAAGTATTGCCAACATCACAATCTT
Pink-footed goose *slc26a12p*	GACCAGGCCGCTTACAAGCAACTCAGCAGTATTGCCAACATCAAAATCTT
Swan goose *slc26a12p*	GACCAGGCCGCTTACAAGCAACTCAGCAGTATTGCCAACATCAAAATCTT
Black swan *slc26a12p*	GACCAGGCTGCTTACAAGCAACTCAGCAGTATTGCCAACATCAAAATCTT

Common snapping turtle *slc26a12*	CAGGTTCGATTCCTCCCTTTACTACGCCAACAAGAGCTACTTCAGGAGCT
Green sea turtle *slc26a12p*	CCGCTTTGAGTCTTTCCTCTACTATGCCAACAAGGACTATTTCAAGGCTG
Dalmatian pelican *slc26a12*	CCGCTTTGAGTCATCCCTCTACTATGCCAACAAGGACTATTTCAAGACTG
Little egret *slc26a12p*	CCGCTTTGAGTCTTTCCTCTACTATGCCAACAAGGACTATTTCAAGGCTG
Emperor penguin *slc26a12p*	--
Rockhopper penguin *slc26a12p*	--
Yellow-eyed penguin *slc26a12p*	--
Adelie penguin *slc26a12p*	--
Magellanic penguin *slc26a12p*	--
Chicken *slc26a12*	CCGCTTTGGGTCGTCCCTTTATTATGCCAACAAAGACTATTTCAAGACTG
Mallard *slc26a12p*	CCGCTTCGAGTCGTCCCTTTACTATGCCAACAAAGATTATTTCAAGACTG
Duck *slc26a12p*	CCGCTTCGAGTCGTCCCTTTACTATGCCAACAAAGATTATTTCAAGACTG
Muscovy duck *slc26a12p*	CCGCTTCGAGTCGTCCCTTTACTATGCCAACAAAGATTATTTCAAGACTG
Pink-footed goose *slc26a12p*	CCGCTTCGAGTTGTCCCTTTACTATGCCAACAAAGATTATTTCAAGACTG
Swan goose *slc26a12p*	CCGCTTCGAGTTGTCCCTTTACTATGCCAACAAAGATTATTTCAAGACTG
Black swan *slc26a12p*	CCGCTTCGAGTTGTCCCTTTACTATGCCAACAAAGATTATTGCAAGACAG

Common snapping turtle *slc26a12*	CGCTCTACCAGAAAACTGGGGTGAATCCTTCCCTGGTGGCTGCCAAACAA
Green sea turtle *slc26a12p*	TTCTTTACCAGAAAACTGGGGTAAACCCTATCCTGCTGGCTGCTAAGCAC
Dalmatian pelican *slc26a12*	TTCTCTACCAGAAAACTGGGGTAAATCCTGTCCTGATGGCTGCTAAGCAC
Little egret *slc26a12p*	TTCTTTACCAGAAAACTGGGGTAAACCCTATCCTGCTGGCTGCTAAGCAC
Emperor penguin *slc26a12p*	--
Rockhopper penguin *slc26a12p*	--
Yellow-eyed penguin *slc26a12p*	--
Adelie penguin *slc26a12p*	--
Magellanic penguin *slc26a12p*	--
Chicken *slc26a12*	CGCTCTACCAGAAAACTGGGGTGAATCCCATCCTGCTGGCTGCCAGGCAT
Mallard *slc26a12p*	TTCTCTACCAGAAAACTGGGTTAAATCCCATCCTGCTGGCTGCTAGGCAT
Duck *slc26a12p*	TTCTCTACCAGAAAACTGGGTTAAATCCCATCCTGCTGGCTGCTAGGCAT
Muscovy duck *slc26a12p*	TTCTCTACCAGAAAACTGGGTTAAATCCCATCCTGCTGGCTGCTAGGCAT
Pink-footed goose *slc26a12p*	TTCTCTACCAGAAAGCTGGGTTAAATCCCACCCTGCTGGCTGCTAGACAT
Swan goose *slc26a12p*	TTCTCTACCAGAAAGCTGGGTTAAATCCCACCCTGCTGGCTGCTAGACAT
Black swan *slc26a12p*	TTCTCTACCAGAAAACTGGGTTAAATCCCACCCTGCTGGCTGCTAGGCAT

Common snapping turtle *slc26a12*	CGAAAGGCTCAATCCAAGACAAAGGCAAACTTGGGCAGCATCCAG-----
Green sea turtle *slc26a12p*	CAAAAGGTGGAGGCCCAGGAAAA---------------------------
Dalmatian pelican *slc26a12*	CAAAGGGTAGAGGCCCAGGCAAATGCAGACATGGGCAACAGCAAGAGCTT
Little egret *slc26a12p*	CAAAAGGTGGAGGCCCAGGAAAA---------------------------
Emperor penguin *slc26a12p*	--
Rockhopper penguin *slc26a12p*	--
Yellow-eyed penguin *slc26a12p*	--
Adelie penguin *slc26a12p*	--
Magellanic penguin *slc26a12p*	--
Chicken *slc26a12*	CAAAGGGCAGTGACCCAGGCAAAAGCAGACACAGGCAACAGGAAGAGCGT
Mallard *slc26a12p*	CAAAGGGTGGAAAGCCAGGCAAATGCAGACACAGGCAATGGTGAGAGATT
Duck *slc26a12p*	CAAAGGGTGGAAAGCCAGGCAAATGCAGACACAGGCAATGGTGAGAGATT
Muscovy duck *slc26a12p*	CAAAGGGTGGAAAGCCAGGCAAATGCAGACACAGGCAACGGTGAGAGATT
Pink-footed goose *slc26a12p*	CAAAGGGTGGAGAGCCAGGCAAATGCAGACATGGGCAATGGCAAGAGATT
Swan goose *slc26a12p*	CAAAGGGTGGAGAGCCAGGCAAATGCAGACATAGGCAATGGCAAGAGATT
Black swan *slc26a12p*	CAAAGGGTGGAGCGCCAGGCAAATGCAGACACAGACAACGGCAAGAGG-T

Common snapping turtle *slc26a12*	---A
Green sea turtle *slc26a12p*	--
Dalmatian pelican *slc26a12*	TTTCGGCACCAGGTTGGACTGCCTGAAACAGGGCACAAAAAGGGCTGAGA
Little egret *slc26a12p*	--
Emperor penguin *slc26a12p*	--
Rockhopper penguin *slc26a12p*	--
Yellow-eyed penguin *slc26a12p*	--
Adelie penguin *slc26a12p*	--
Magellanic penguin *slc26a12p*	--
Chicken *slc26a12*	TTTTAATTCTGTGTTTGGTTGCCTGAAACCTTCCAAGAAACACATGGGGA
Mallard *slc26a12p*	TTTTGGCACTGGGTTTGACTGCCTGAAACCTGCCAAGACGAGGGCCGAGA
Duck *slc26a12p*	TTTTGGCACTGGGTTTGACTGCCTGAAACCTGCCAAGACGAGGGCCGAGA
Muscovy duck *slc26a12p*	TTTTGGCACTGGGTTTGACTGCCTGAAACCTGCCAAGACAAGGGCTGAGA
Pink-footed goose *slc26a12p*	TTTTGGCACCGGGTTTGACTGCCTGAAACCTGCCAAGACAAGGGCCGAGA
Swan goose *slc26a12p*	TTTTGGCACCGGGTTTGACTGCCTGAAACCTGCCAAGACAAGGGCCGAGA
Black swan *slc26a12p*	TTTTGGCACCAGGTTTGACTGCCTGAAACCTGCCAAGACAAGGGCCGAGA

Common snapping turtle *slc26a12*	AGGACTCAACAGCAGTCGCCATTCCGCCAATAGATATGCACACCTTAGTC
Green sea turtle *slc26a12p*	-GCCTCCAGCAGATGCCTGTCCTCCCTCCTTAGATATGCACACCTTAATC
Dalmatian pelican *slc26a12*	AGCCTCCAGCAGATGCCTGTCCTCCCTCCACAGATATGCACACCTTAATC
Little egret *slc26a12p*	-GCCTCCAGCAGATGCCTGTCCTCCCTCCTTAGATATGCACACCTTAATC
Emperor penguin *slc26a12p*	--
Rockhopper penguin *slc26a12p*	--
Yellow-eyed penguin *slc26a12p*	--
Adelie penguin *slc26a12p*	--
Magellanic penguin *slc26a12p*	--
Chicken *slc26a12*	AATCGCCCACAGACGTCTGCCTGCCCTCCCTAGATATGCACACTTTAATC
Mallard *slc26a12p*	AGTCACCCCCAGATGTCTGCCCTCCCTCCGTAGATACACACACCTTAATC
Duck *slc26a12p*	AGTCACCCCCAGATGTCTGCCCTCCCTCCGTAGATACACACACCTTAATC
Muscovy duck *slc26a12p*	AGTCACCCCCAGATGTCTGCCCTCCTTCCGTAGATACACACACCTTAATC
Pink-footed goose *slc26a12p*	AGTCACCCCCAGATGTCTGTCCTCCCTCTGTAGATAAGCACACCTTAATC
Swan goose *slc26a12p*	AGTCACCCCCAGATGTCTGTCCTCCCTCTGTAGATAAGCACACCTTAATC
Black swan *slc26a12p*	AGTCACCCCCAGATGTCTGCCCTCCCTCTGTAGATACGCACGCCTTAATC

Common snapping turtle *slc26a12*	ATTGACTGTGGGGCCATGCAGTTCTTGGACACCGTGGGCCTCAGCGTGCT
Green sea turtle *slc26a12p*	CTTGACTGTGGGGCAATGCAGTTCATAGATACTGTGGGTCTCTCTGTGCT
Dalmatian pelican *slc26a12*	CTTGACTGTGGGGCAATGCAGTTCATAGATACCATGGGTCTCTCTGTGCT
Little egret *slc26a12p*	CTTGACTGTGGGGCAATGCAGTTCATAGATACTGTGGGTCTCTCTGTGCT
Emperor penguin *slc26a12p*	----------------------------TTGTGGTGGCTTGCGTTGTG--
Rockhopper penguin *slc26a12p*	--
Yellow-eyed penguin *slc26a12p*	--
Adelie penguin *slc26a12p*	--
Magellanic penguin *slc26a12p*	--
Chicken *slc26a12*	CTTGACTGTGGGGCTATGCAGTTCATAGATACCACGGGTCTCTCCGTGCT
Mallard *slc26a12p*	ATTGACTGTGGGGCGATGCAGTTCATAGATACCGTGGGTTTCTCCATGTT
Duck *slc26a12p*	ATTGACTGTGGGGCGATGCAGTTCATAGATACCGTGGGTTTCTCCATGTT
Muscovy duck *slc26a12p*	ATTGACTGTGGGGCGATGCAGTTCATAGATACCGTGGGTTTCTCCATGTT
Pink-footed goose *slc26a12p*	ACTGACTGTGGGGCGATGCAGTTCATAGATACCGTGGGTCTCTCCATGCT
Swan goose *slc26a12p*	ACTGACTGTGGGGCGATGCAGTTCATAGATACCGTGGGTCTCTCCATGCT
Black swan *slc26a12p*	ATTGACTGTGGGGCGATGCAGTTCATAGATACCGTGGGTCTCTCCGTGCT

Common snapping turtle *slc26a12*	GAAGGAGATGCGCCATGATTACAGGAAGATTGGCATCCAGGTCTTATTGG
Green sea turtle *slc26a12p*	GAAGGAGACACATCATGACTATAAGGAGGTTGGCATCCAGGTGCTCTTGG
Dalmatian pelican *slc26a12*	AAAGGAGACACATCAGGACTATAAGAAGATTGGTGTCCAGGTGCTCCTGG
Little egret *slc26a12p*	GAAGGAGACACATCATGACTATAAGGAGGTTGGCATCCAGGTGCTCTTGG
Emperor penguin *slc26a12p*	--
Rockhopper penguin *slc26a12p*	--
Yellow-eyed penguin *slc26a12p*	--
Adelie penguin *slc26a12p*	--
Magellanic penguin *slc26a12p*	--
Chicken *slc26a12*	GAAAGAGACGCACCGTGACTTCCAGGAGCTCGGCGTCCAGCTGCTCCTGG
Mallard *slc26a12p*	GAAGGACACACATCATGACAACAAGGAGATTGGTGTCCAGGTGCTCCTGG
Duck *slc26a12p*	GAAGGACACACATCATGACAACAAGGAGATTGGTGTCCAGGTGCTCCTGG
Muscovy duck *slc26a12p*	GAAGGACACACATCATGACAACAAGGAGATTGGTGTTCAGGTGCTCCTGG
Pink-footed goose *slc26a12p*	GAAGGACACACATCACGACTACAAGGAGATTGGTGTCCAGGTGCTCCTGG
Swan goose *slc26a12p*	GAAGGACACACATCACGACTACAAGGAGATTGGTGTCCAGGTGCTCCTGG
Black swan *slc26a12p*	GAAGGACACACATCACGACTACAAGGAGATTGGTGTCCAGGTGCTCCTGG

Common snapping turtle *slc26a12*	CCAATTGCAACCCTTCCATCCG----TCGCCTGCTCCAGGATGGGGGTTG
Dalmatian pelican *slc26a12*	CCAACTGCAACCCTTCCATCCG----CCACCGGCTCCGGGAGGGAGGCTG
Green sea turtle *slc26a12p*	CCAACTGCAACCCTTCTATCTG----CTGCCAGCTCCGGGAGGGAGGCTG
Little egret *slc26a12p*	CCAACTGCAACCCTTCTATCTG----CTGCCAGCTCCGGGAGGGAGGCTG
Emperor penguin *slc26a12p*	--
Rockhopper penguin *slc26a12p*	--
Yellow-eyed penguin *slc26a12p*	--
Adelie penguin *slc26a12p*	--
Magellanic penguin *slc26a12p*	--
Chicken *slc26a12*	CCAACTGCAACCCTCTCCTC----CGCCGCCGGCTGCGGGATGGTGGTTG
Mallard *slc26a12p*	CCAACTGCAGCCCTTCCATC----CGCCAC--------------------
Duck *slc26a12p*	CCAACTGCAGCCCTTCCATC----CGCCAC--------------------
Muscovy duck *slc26a12p*	CCAACTGCAGCCCTTCCATC----CGCCACTG------------------
Pink-footed goose *slc26a12p*	CCAACTGTAACCCTTCTACTGTAACGCCACCGGCTCCGGGCGGGAGGCTG
Swan goose *slc26a12p*	CCAACTGTAACCCTTCTACTGTAACGCCACCGGCTCCGGGCGGGAGGCTG
Black swan *slc26a12p*	CCAACTGTAACCCTTCTATC----CGCCACTGGCTCCGGGCGGGAGGCTG

Common snapping turtle *slc26a12*	GGAGATTGAGACAG---GAAATGGTGAGCTGGCCTTCCACAGTGTCCACG
Green sea turtle *slc26a12p*	GGCTGGCAAGACAGACAGTGGTGGTCAGTTGGCTTTCCACAGTGTCCATG
Dalmatian pelican *slc26a12*	GTCTGGCGAGACAAACAGTGGCGGTCAGCTGGCTTTCCACAGCATCCACA
Little egret *slc26a12p*	GGCTGGCAAGACAGACAGTGGTGGTCAGTTGGCTTTCCACAGTGTCCATG
Emperor penguin *slc26a12p*	------------------------TCAGCTGGCTTTCCACAGCGTCCATG
Rockhopper penguin *slc26a12p*	------------------------TCAGCTGGCTTTCCACAGCGTCCATG
Yellow-eyed penguin *slc26a12p*	-----------------------GTCAGCTGGCTTTCCACAGCGTCCATG
Adelie penguin *slc26a12p*	------------------------TCAGCTGGCTTTCCACAGCATCCATG
Magellanic penguin *slc26a12p*	-----------------------GTCAGCTGGCTTTCCACAGCGTCCATG
Chicken *slc26a12*	GGCTGCCGGGGCG---CACGGCGGCCAGCTGGCTTTCCACAGCGTGCACC
Mallard *slc26a12p*	--AGCGTCC-TG
Duck *slc26a12p*	--AGCGTCC-TG
Muscovy duck *slc26a12p*	--------------------------------CTTTCCACAGCGTCC-TG
Pink-footed goose *slc26a12p*	GGCTGGCCAGGCAGCTAGCGGTGGGCAGCTGGCTTTCCACAGCGTCCATG
Swan goose *slc26a12p*	GGCTGGCCAGGCAGCTAGCGGTGGGCAGCTGGCTTTCCACAGCGTCCATG
Black swan *slc26a12p*	GGCTG--------------GCCAGGCAGCTGGCTTTCCACAGCGTCCACG

Common snapping turtle *slc26a12*	ATGCTGTGCAGTTTGCAGAGAGTCAGTACCAAGAGCAGCACAAGGACAGC
Green sea turtle *slc26a12p*	ATGCCGTGCAGTTTGCTGAACAGTGGTACCATGTGCAGCAGGAGGAGAGC
Dalmatian pelican *slc26a12*	ATGCAGTGCAGTTTGCTGAACAGTCCTACCATGTGCAGCAGGAGGAGAGC
Little egret *slc26a12p*	ATGCCGTGCAGTTTGCTGAACAGTGGTACCATGTGCAGCAGGAGGAGAGC
Emperor penguin *slc26a12p*	ATGCAGTGCAGTTTGCTGAACGGTGGTACTATGTGCAGCAGGAGGAGAGC
Rockhopper penguin *slc26a12p*	ATGCAGTGCGGTTTGCTGAACGGTGGTTCTATGTGCAGCAGGAGGAGAGC
Yellow-eyed penguin *slc26a12p*	ATGCAGTGCGGTTTGCTGAACGGTGGTACTATGTGCAGCAGGAGGAGAGC
Adelie penguin *slc26a12p*	ATGCAGTGCAGTTTGCTGAACGGTGGTACTATGTGCAGCAGGAGGAGAGC
Magellanic penguin *slc26a12p*	ATGCAGTGCGGTGTGCTGAACGGTGGTACTATGTGCAGCAGGAGGAGAGC
Chicken *slc26a12*	ACGCGGTGCAGTTTGCACAGCAGTGGCACCGCGAGCAGCAGGAG---AGC
Mallard *slc26a12p*	ATGCAGCGCAGGTTGCTGAGCAGTGGTATCAGGTGCATCAAGAG---AGC
Duck *slc26a12p*	ATGCAGCGCAGGTTGCTGAGCAGTGGTATCAGGTGCATCAAGAG---AGC
Muscovy duck *slc26a12p*	ATGCAGCGCAGGTTGCTGAGCAGTGGTATCAGGTGCATCAAGAG---AGC
Pink-footed goose *slc26a12p*	ATGCAGTGCAGTTTGCTGAGCAGTGGTACCG--TGCAGCAAGAG---AGC
Swan goose *slc26a12p*	ATGCAGTGCAGTTTGCTGAGCAGTGGTACCG--TGCAGCAAGAG---AGC
Black swan *slc26a12p*	ATGCAGTGCAGTTTGCTGAGCAGTGGTACCG--TGTAGCAAGAG---AGC

Common snapping turtle *slc26a12*	GAGGAGGTAGGGGCTGCTTTCGTTGACAATGAGGTTCAGAACATCGCAGA
Green sea turtle *slc26a12p*	AAGGAGAAGAAGGATGCTCTCCTG------------------GACACCGA
Dalmatian pelican *slc26a12*	AAGGAGAAGAAGAATGCTCTCCTG------------------GACCCCGA
Little egret *slc26a12p*	AAGGAGAAGAAGGATGCTCTCCTG------------------GACACCGA
Emperor penguin *slc26a12p*	AAGGAGCAAAAGGATGCTGTCCAG------------------GACCCCGA
Rockhopper penguin *slc26a12p*	AAGGAGAAAAAGGATGCTGTCCTG------------------CACCCCGA
Yellow-eyed penguin *slc26a12p*	AAGGAGAAAAAGGATGCTGTCCTG------------------CACCCCGA
Adelie penguin *slc26a12p*	AAGGAGAAAAAGGATGCTGTCCTG------------------GACCCCGA
Magellanic penguin *slc26a12p*	AAGGAGAAAAAGGATGCTGTCCTG------------------CACCCCGA
Chicken *slc26a12*	AAGGAGAGAAGGGATGCTGACCCG------------------GACCCCGA
Mallard *slc26a12p*	AGGGAGAAAAAGGATGCGCTCCTG------------------GACCCTGA
Duck *slc26a12p*	AGGGAGAAAAAGGATGCGCTCCTG------------------GACCCTGA
Muscovy duck *slc26a12p*	AGGGAGAAAAAGGATGCGCTCCTG------------------GACCCTGA
Pink-footed goose *slc26a12p*	AGGGAGAAAAAGGATGCGCTCCTG------------------GACCCCGA
Swan goose *slc26a12p*	AGGGAGAAAAAGGATGCGCTCCTG------------------GACCCCGA
Black swan *slc26a12p*	AGGGAGAAAAAGGATGTGCTCCTG------------------GACCCCGA

Common snapping turtle *slc26a12*	GGGCCCCAATATGGAGACAGCTCTATAA
Green sea turtle *slc26a12p*	AGACTCAAACTTCCAGTCGTCTCTGTAG
Dalmatian pelican *slc26a12*	AGACCTGAACTTCCAGGTGTCTTTGTAG
Little egret *slc26a12p*	AGACTCAAACTTCCAGTCGTCTCTGTAG
Emperor penguin *slc26a12p*	AGACCTGAACTTCCAGGCATCTTTGTAG
Rockhopper penguin *slc26a12p*	AGACCTGAACTTCCAGGTGTCTTTGTAG
Yellow-eyed penguin *slc26a12p*	AGACCTGAACTTCCAGGTGTCTTTGTAG
Adelie penguin *slc26a12p*	AGACCTGAACTTCCAGGTGTCTTTGTAG
Magellanic penguin *slc26a12p*	AGACCTGAACTTCCAGGTGTCTTTGTAG
Chicken *slc26a12*	GGACATGAGCGTCCAAGCGTCTCTGTAG
Mallard *slc26a12p*	AGACCCACACGTCCAGACGTCTTTGTAG
Duck *slc26a12p*	AGACCCACACGTCCAGACGTCTTTGTAG
Muscovy duck *slc26a12p*	AGACCCGCACATCCAGACGTCTTTGTAG
Pink-footed goose *slc26a12p*	AGACCCGCATGTCCAGACATCTTTGTAG
Swan goose *slc26a12p*	AGACCCGCATGTCCAGACATCTTTGTAG
Black swan *slc26a12p*	AGGCCTGCACGTCCAGATATCTTTGTAG

Figure S2. Multiple alignment of exons of *slc26a12* and *slc26a12p* in turtles and birds. Deletions, insertions, and mutations that contribute to pseudogenization of *slc26a12* are indicated in red.
